# Nutrition and Physical Activity-Induced Changes in Gut Microbiota: Possible Implications for Human Health and Athletic Performance

**DOI:** 10.3390/foods10123075

**Published:** 2021-12-10

**Authors:** Vittoria Cella, Viviana M. Bimonte, Claudia Sabato, Antonio Paoli, Carlo Baldari, Matteo Campanella, Andrea Lenzi, Elisabetta Ferretti, Silvia Migliaccio

**Affiliations:** 1 Department of Movement, Human and Health Sciences, University Foro Italico, 00135 Rome, Italy; cellavittoria@outlook.it (V.C.); viviana.bimo@gmail.com (V.M.B.); 2 Department of Experimental Medicine, University Sapienza, 00161 Rome, Italy; claudia.sabato@uniroma1.it (C.S.); andrea.lenzi@uniroma1.it (A.L.); elisabetta.ferretti@uniroma1.it (E.F.); 3 Department of Biomedical Sciences, University of Padua, 35143 Padua, Italy; antonio.paoli@unipd.it; 4 Department of Theoretical and Applied Sciences, eCampus University, 22060 Novedrate, Italy; carlo.baldari@uniecampus.it (C.B.); matteo.campanella93@gmail.com (M.C.)

**Keywords:** gut microbiota, microbial composition, health, exercise, athletes, physical performance, hormones

## Abstract

The gut microbiota is a complex heterogeneous microbial community modulated by endogenous and exogenous factors. Among the external causes, nutrition as well as physical activity appear to be potential drivers of microbial diversity, both at the taxonomic and functional level, likely also influencing endocrine system, and acting as endocrine organ itself. To date, clear-cut data regarding which microbial populations are modified, and by which mechanisms are lacking. Moreover, the relationship between the microbial shifts and the metabolic practical potential of the gut microbiota is still unclear. Further research by longitudinal and well-designed studies is needed to investigate whether microbiome manipulation may be an effective tool for improving human health and, also, performance in athletes, and whether these effects may be then extended to the overall health promotion of general populations. In this review, we evaluate and summarize the current knowledge regarding the interaction and cross-talks among hormonal modifications, physical performance, and microbiota content and function.

## 1. Introduction

It has been largely demonstrated that a wrong nutrition and a sedentary lifestyle are linked to a high incidence of chronic metabolic diseases such as cardiovascular disease (CVD), type 2 diabetes mellitus (T2DM), cancer and osteoporosis [1], while exercise plays a fundamental role in preventing and treating these pathologies [2]. Interestingly, the health-promoting actions of exercise are mediated by metabolic and immune effects that involve several mechanisms, such as the promotion of an anti-inflammatory state, activation of the hypothalamic–pituitary–adrenal (HPA) axis, augmentation of synaptic plasticity and reinforcement of neuromuscular function [3,4]. Moreover, skeletal muscle acts like an endocrine organ by producing a multitude of hormones and cytokines in response to muscle contraction, which exert their impacts on several organs and tissues [5]. In recent years, scientific research has been considered the gut microbiota as a new potential target by which physical activity might impact health status, gut microbiome profile, in terms of quali/quantitative features (i.e., microbial richness, presence/absence, or number of certain taxa) since it is not a rigid trait, but instead it reacts to environmental and life-style factors [6]. Alterations of the gut bacterial community, both at the taxonomic and functional level, have been linked with many diseases, from obesity [7] and metabolic disorders [8] to brain-related dysfunctions [9,10]. Interestingly, various studies have demonstrated that the manipulation of gut microbiota and its by-products through exercise prescription might represent a promising novel approach in preventing and treating metabolic pathological conditions [11,12,13,14]. Moreover, several studies suggest that gut microbiome modification (i.e., abundance of health-promoting bacterial species, increased microbial diversity) in response to exercise may provide insights for improving athletic performance and/or recovery time after training [15,16,17,18]. This review will evaluate latest findings on exercise-induced alterations of the gut microbiota both in animals and humans, with a particular focus on physical performance and athlete populations.

## 2. Gut Microbiota: Composition, Characterization and Function

The human gut microbiota is a complex system of mutualistic microorganisms that live all through the gastrointestinal tract (GI tract), increasing in number and diversity from the stomach to the colon. It consists of almost 39 trillion microbes, which is a 1:1 ratio of microbial to eukaryotic cells in the human body, over 1000 unique bacterial species and over 3 million unique genes [19,20]. Along with bacteria, the gut microbiota also houses other prokaryotes (i.e., Archaea), fungi, and viruses (10). In healthy adult subjects, two main bacterial phyla are prevalent: Gram-positive Firmicutes (ranging from 60% to 80%) and the Gram-negative Bacteroidetes (ranging from 15% to 30%) [21,22]. The Firmicutes phylum contains over 250 genera of bacteria, including *Lactobacillus* and *Clostridium*, while Bacteroidetes phylum contains nearby 20 genera, the most abundant being *Bacteroides* [23], while the minority bacteria belong to the Proteobacteria, Actinobacteria, Fusobacteria, and Verrucomicrobia phyla [24]. With the advent of next-generation DNA sequencing, the culture-based methodologies for gut microbes studies were replaced by culture-independent genomic analysis of microbiota, the most applied of which is based on the amplicon sequencing of the 16S ribosomal ribonucleic acid (rRNA) gene in archaea and bacteria [18,25]. This technique allows the taxonomical classification of bacteria at the genus or species level [26], thus giving a general idea of the taxonomic composition of a microbial ecosystem [18,27]. In particular, this technique is different from amplicon sequencing, as it produces sequences from random fragments of the whole microbiota DNA [27] and, thanks to advances in allying complete metagenome-assembled genomes with shotgun analyses, it permits to detect bacteria taxa even at the strain level [28], the lower level of taxonomic classification describing genetic variants or subtypes of a species [18]. The central role of microbiota in human health maintenance and homeostasis depends on its numerous protective, metabolic, and structural functions [29] (Table 1), exerted by the production and release of various molecules, such as amino acids, short-chain fatty acids (SCFAs) and regulatory enzymes. 

In fact, the microbiome is involved in digestion functions, biosynthesis and absorption of nutrients, maintenance of epithelial integrity and mucosal homeostasis, interface with immune system, competitive inhibition of conceivable invasion and colonization by pathogenic microorganisms, drugs and xenobiotics metabolism [39,40,41,42].

## 3. Gut Microbiota Produces Molecules with Endocrine Activity

Beyond the local gastrointestinal functions, the gut microbiota exerts its effects also on distal organs and systems, by releasing in the bloodstream molecules that act as hormones [43,72]; this interaction is bidirectional, indeed specific members of the overall microbial community could be modulated by hormones secreted by the host (i.e., stress and catabolic hormones) as suggested by a new area of study termed Microbial Endocrinology [73,74,75]. For this reason, microbiota is now considered as a virtual complex endocrine organ [35,43], that influences host metabolism and energy homeostasis, by regulating several functions including insulin sensitivity, fat storage, adiposity and body weight [15], ultimately affecting host’s health and disease. Moreover, microbiota itself influenced by host endocrine secretions. 

### 3.1. Short Chain Fatty Acids 

Among the microbial metabolites with endocrine activity, a key role is played by short chain fatty acids (SCFAs), predominantly acetate, propionate, and butyrate, whose production and absorption occurs mainly in the proximal large intestine, where colonic bacteria including *Bacteroides*, *Bifidobacterium*, *Propionibacterium*, *Eubacterium*, *Lactobacillus*, *Clostridium*, *Roseburia* and *Prevotella* ferment undigested food [35,76]. Acetate and propionate are mostly absorbed from the colonic lumen and transported in the bloodstream to a variety of different organs (i.e., skeletal muscle, adipose tissue, liver) where they serve as substrates for energy metabolism [77,78], while n-butyrate is the substrate of colonocytes [79,80]. The latter has an impact on numerous functions, such as colon cancer and inflammatory bowel disease protection, immune modulation, intestinal barrier regulation, oxidative stress decrease [62,63]. In skeletal muscle, butyrate may play a pivotal role in activating several regulatory pathways resulting in increased ATP production and improved metabolic efficiency of myofibers [60], and it also inhibits histone deacetylase, preventing apoptosis and protecting against muscle protein catabolism [61]. SCFAs represent the key hormonal mediators of the gut microbiota, they can activate specific receptors, such as G-protein coupled receptors, GPR43 and GPR41 [30], expressed in several different tissues and cell types (i.e., endocrine cells, adipocytes, skeletal muscle cells) [30,31,32,33,34]. For instance, SCFA-GPR43 signaling might contribute to regulate immune and inflammatory responses, including intestinal inflammation [53], by controlling neutrophil chemotaxis [54] and by acting on the proliferation of T regulatory cells [55]. GPR43 may influence adipocyte function as suggested by its induction during adipocyte differentiation as well as by its increase during high-fat diet in rodents [81]. Moreover, an effect on lipid accumulation and inhibition of lipolysis by acetate and propionate, mainly through GPR43, has been demonstrated by Hong et al. [82], while GPR41/GPR43 have been associated with regulation of host adipose tissue and leptin production by the gut microbiota [68,69,70]. GPR41 and GPR43 are expressed in enteroendocrine L-cells in ileum and colon intestinal portions [71], and activation of these receptors by SCFAs promotes the secretion of the anorexigenic gut peptide glucagon-like peptide-1 (GLP-1) and peptide YY (PYY), as shown by several reports [71,77,83], thus regulating food intake and appetite, gut barrier and glucose homeostasis (i.e., insulin sensitivity) via direct interactions with organs but also through nervous routes [66]. The modulation of glucose uptake and metabolism, as well as the promotion of insulin sensitivity, occurs also in skeletal muscle cells [67], where SCFAs derived from the systemic circulation bind to both GPR41 and GPR43 receptors [33,34]. Interestingly, it has been shown that GPR41 is highly expressed in rat brain tissue [67], and monocarboxylate transporters used by SCFA are abundantly expressed at the blood-brain barrier [84,85,86,87], thus suggesting that circulating SCFAs could cross the blood-brain barrier, thus entering the central nervous system (CNS), where they are thought to play a role as major energy source in cellular metabolism [38], neutrophil cells signaling [45,46,47] and neurotransmitter synthesis [48].

### 3.2. Neurotransmitters

Moreover, gut microbiota is itself a source of local neurotransmitters, influencing the HPA axis, thus affecting mood, motivation, and sensation of exhaustion in both sedentary individuals and athletes [49]. These molecules include nitric oxide [50,51], GABA, produced by several *Lactobacillus* and *Bifidobacterium* with large interspecies variation [88], and monoamines such as noradrenaline, dopamine and serotonin, produced by certain strains of bacteria [89,90]. Additionally, the circulating concentrations of the aminoacid tryptophan, a precursor of serotonin, a key neurotransmitter in the gut-brain axis both at the enteric level [91] and the central nervous system [52], are controlled by the bacteria in the gut. Strenuous training and competition might cause mood disturbances, fatigue, insomnia, and depression in athletes, ultimately affecting physical performance. Moreover, the production and modulation of different neurotransmitters and hormones by gut microbes might also contribute to behavior regulation [49,52]. Interestingly, tryptophan could also stimulate insulin-like growth factor 1/Ribosomal protein S6 kinase β-1 (p70S6K)/mammalian target of the rapamycin (mTOR) pathway in muscle cells, promoting the expression of genes involved in myofibrillar synthesis [56].

### 3.3. Secondary Bile Acids

Other microbial by-products with relevance in host metabolism include secondary bile acids, which affect glucose homeostasis by activating receptors similar to those activated by the parent compounds (e.g., farnesoid X receptor-α) [43], and trimethylamine, derived by choline degradation [44]. Trimethylamine conversion to trimethylamine-N-oxide is involved in cardiovascular disease [92,93] and choline deficiency is related to nonalcoholic fatty liver disease and altered glucose metabolism [8,94,95]. 

## 4. Endogenous and Exogenous Factors Influence Gut Microbiota

Composition of the gut microbiota communities shows high interindividual variability, especially at levels below the phylum [96]. Host-specificity is influenced by several endogenous and exogenous determinants, including host genetics [36,97], gender [98], geographic origin [99,100], pregnancy [101], type of birth (natural or caesarian) [102], method of infant feeding (breastfeeding or infant formula) [103], stress, drugs [104] and diet (Table 2).

Indeed, dietary habits strongly impact gut microbiota composition. Thus, western diets, characterized by a high content of sugars and fats and low content of fiber, have been linked with a decrease in community diversity, permanent loss of bacteria and dysbiosis [105,106,107]. Conversely, high fiber diets, including fruits, vegetables, legumes, and whole-wheat grain products, can increase microbial diversity [108,109]. Shifts in the microbial community in response to different factors impair the symbiotic relationship between pathogenic and nonpathogenic bacteria, potentially causing the onset of a proinflammatory state, and gut dysbiosis, with health implications [112], including autoimmune and allergic conditions, colorectal cancer, metabolic diseases [9,113,114]. Although gut microbiota alterations have been identified as contributing factors of different host diseases, the existence of “health-associated” microbial profiles, is still unknown, despite some bacterial species, have been suggested as crucial for the development of a healthy microbiota [20,96,115]. Increased gut microbial species diversity and/or richness are recognized as features of healthy individuals [64] showing a positive association with overall gut microbial richness. Interestingly, Roager et al. have observed that human slower colonic transit time, assessed by radiopaque markers, was strongly related to a shift in colonic metabolism from carbohydrate fermentation to protein catabolism while shorter colonic transit time appeared linked to metabolites possibly reflecting increased renewal of the colonic mucosa. Together, these data indicate that gut microbial diversity may not be the only determinant of a healthy gut ecosystem, but other factors, such as colonic transit time, should be considered [116]. In addition, it seems that gene content/diversity in the intestinal tract might be a better biomarker of physiological states [117] and, therefore, the functional/metabolic activity of gut microbiota may have a greater impact than microbial composition on the onset and/or maintenance of human health or disease development.

Impact of Exercise on Gut Microbiota

**Figure 1:**
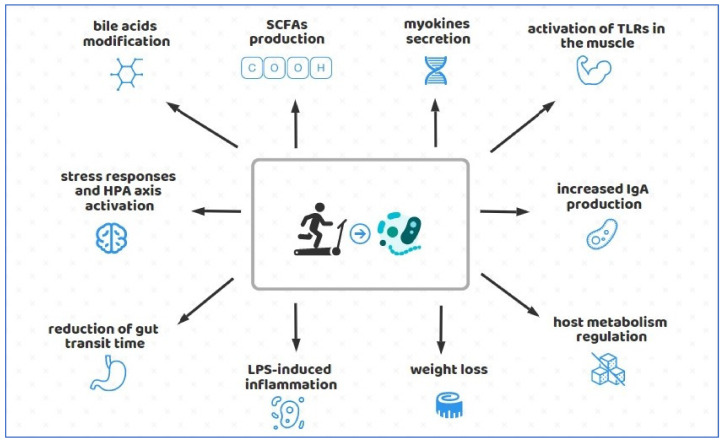
Schematic representation of the effect of physical activity on gut microbiota. Physical activity could alter the gut microbiota through several pathways, as depicted in figure. Specifically, the mechanisms involved in the effects are the pathways involved in modification of bile acid profiles, SCFAs production, myokines secretion, activation of Toll-Like Receptors (TLRs) in the muscle by LPS, increase IgA production, glucose homeostasis maintenance, HPA axis activation. Moreover, physical activity influence also weight loss, exercise-induced heat stress, reduction of gut transient time.

Physical activity prescription plays a crucial role in the prevention of several diseases, such as CVD, colon, breast cancer, T2DM, osteoporosis, sarcopenia, cognitive impairment, and depression [118]. Within the last years, it has been largely reported that exercise-related benefits on GI tract, metabolic diseases, mood, and other brain-related disorders could be provided by alterations to the gut microbial community and its metabolites [11,13,14,43,62,110,111]. Although various studies have shown that physical activity increases microbiota diversity and modulates its distribution and functional capacity [15,16,17,119], the results are still conflicting. Furthermore, evidence on the relationship between exercise, gut permeability and lipopolysaccharide (LPS)-induced systemic inflammation are not conclusive. Discrepancies among studies could depend on the type, intensity, duration, and adaptability of the exercise applied [57]. Indeed, it has not yet been clarified whether an acute systemic elevation of LPS in response to prolonged or strenuous exercise, often referred as “mild endotoxemia” [120,121,122], might occur as a physiological transient effect or might be detrimental, in the long term, to health, particularly in leisure athletes who regularly practice physical activity [121,123]. Moreover, the mechanisms by which exercise could cause changes in the microbiota are not yet fully understood, likely involving a set of interrelated pathways, summarized in the Figure 1 including, for instance, modification of bile acid profiles, increased SCFAs production, activation of toll-like receptors (TLRs) in the muscle by LPS, myokines release from muscle fibers, glucose homeostasis maintenance, weight loss induced by energy expenditure, exercise-induced heat stress, HPA axis activation and other under recognized contributing factors (Figure 1) [96,124,125]. 

Of particular interest is the notion that the effects of exercise on gut microbial shifts might also be driven by hormonal signals. Psychological and physical demands associated with prolonged intense exercise and inadequate recovery could lead to a stress response that involves activation of sympathetic adrenomedullary and HPA axes, leading to the release of stress and catabolic hormones [126], inflammatory cytokines (i.e., TNFα, INFα, INFγ), interleukins (IL1β or IL6), and gut microbiota molecules (i.e., SCFAs, tryptophan, serotonin, GABA, dopamine). Indeed, recent evidence in experimental murine models suggests a high correlation between physical and emotional stress during exercise and changes in gastrointestinal microbiota composition [49,127]. Furthermore, in vitro studies have shown a growth of non-pathogenic commensal *E. coli* [128], as well as of other Gram-negative bacteria [129], in response to increased noradrenaline concentrations, after acute stress. Noradrenaline may stimulate pathogenic bacterial growth by facilitating the adhesion of E. coli to the intestinal wall by increasing its virulence factor K99 pilus adhesin and by activating the expression of virulence-associated factors [130]. Moreover, a recent study conducted by Karl et al. investigated the effects of physiological stress on intestinal microbiota composition and metabolic activity, as well as intestinal permeability in soldiers submitted to a multiple-stressor military training exercise (4-day cross-country ski-march). Although the findings are obtained in a small population, the results showed an increase in intestinal permeability associated with alterations in inflammatory markers and with changes in microbiota composition and metabolism [58]. Alterations of the gut microbiota, possibly induced by acute and chronic stress hormones released after exercise, have not been explored sufficiently, and should be further investigated to elucidate whether microbial changes may impact on metabolic function and physical performance of both athletes and leisure exercising subjects. 

## 5. Studies in Murine Models

**Figure 2:**
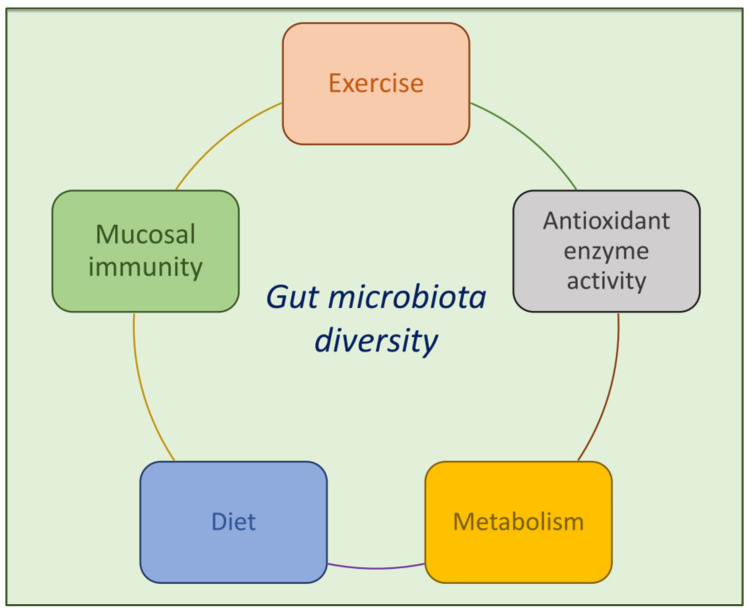
Schematic diagram depicting the factors that contribute to modify the gut microbiota in murine model.

Studies on the impact of exercise on gut microbiota composition and functional capacity in experimental rodent models are not definitive, as also stated by a recent systematic review that highlighted a lack of results supporting a role for exercise in modifying specific taxonomic groups or indices of richness or diversity. Potential causes include differences in study design (i.e., mode, intensity, duration of exercise, diet protocols, species/strain, and age of animal models), statistical indices used for diversity assessment (i.e., Shannon vs. Chao1 vs. QIIME calculated, etc.) and inconsistency in reporting. Only a tendency for an exercise-induced increase of butyrate producing bacteria was detected [131]. A study carried out by Matsumoto et al. showed that voluntary exercise (running) in rats determined a variation in microbiota composition, with a subsequent increase of n-butyrate concentration [62]. These effects were partly confirmed by Queipo-Ortuño et al. that demonstrated how a short period of voluntary wheel running (6 days) was able to increase bacteria producing lactic acid *Lactobacillus* and *Bifidobacterium*, which modulate mucosal immunity and prevent pathogen invasion, and Blautiacoccoides–Eubacteriumrectale group, which convert lactate into butyrate. Moreover, a positive link between *Lactobacillus* and *Bifidobacterium* genera and serum leptin levels was observed in this study [132]. In 2018, Allen et al. confirmed an increased cecalbutyrate: acetate ratio, along with increased abundance of *Akkermansia* and of an unclassified genus within the family *Lachnospiraceae* in exercised mice compared to sedentary controls [133]. In addition, a higher abundance of Firmicutes phylum, in particular of *Lactobacillales* order, was found in exercised mice versus sedentary controls. In particular, this study investigated the effects of polychlorinated biphenyls (PCBs) and 5-weeks of exercise on the composition and structure of the gut microbiome in aged mice showing that the structure of the gut microbiota of exercised mice was significantly different compared to sedentary controls. Furthermore, the overall abundance of bacteria was significantly diminished in PCB-exposed sedentary mice. However, no statistical differences in bacterial community structure were observed in the exercised mice before and after PCB treatment, suggesting that exercise prevented the PCB-induced decrease of Proteobacteria observed in sedentary mice [134]. Moreover, Petriz et al. reported an increase of Firmicutes abundance in gut microbiota of obese rats after moderate exercise training, with higher relative abundance of *Lactobacillus*, suggesting a possible therapeutic role of exercise-induced microbiome modifications in obesity treatment [11]. Conversely, Evans et al. showed that the prevention of weight gain in mice fed a high-fat diet-induced obesity by exercise was associated with enhanced gut biodiversity and a relative increase in Bacteroidetes. The increase of Bacteroidetes: Firmicutes ratio was proportional to the distance run. Moreover, exercise augmented the relative proportion of butyrate-producing bacteria such as the Bacteroidales S24-7 family of Bacteroidia class within the Bacteroidetes phylum, and *Clostridiaceae*, *Lachnospiraceae* and *Ruminococcaceae* of the Clostridia class within the Firmicutes phylum [12]. However, conflicting results were produced by Kang et al. evaluating the gut microbiota of mice on either a high-fat diet or on normal-diet, both conducted with and without forced wheel running exercise did not counteract microbiota changes induced by a high-fat diet [110]. Another study, performed by Campbell et al., examined the effect of 12-week voluntary exercise on intestinal integrity and gut microbial ecology of sedentary and exercised animals on normal and high-fat diets. Clonal and pyrosequencing analyses showed few Bacterioides family members in fecal microbiota, whereas Clostridiales were predominant in all animal groups [135]. The effects of exercise on gut microbiota shifts in T2DM were investigated by Lambert et al. that evaluated cecal microbiota of T2DM (db/db) and control (db/+) mice engaged in 6 weeks of sedentary or low-intensity treadmill running. The study revealed that total bacteria and Enterobacteriaceae were similar in db/+ mice regardless of exercise, but they were lower in exercising db/db mice, and *Bifidobacterium* spp. was greater in exercised non-diabetic mice, while the presence of diabetes nullified this effect [136]. Another interesting aspect was investigated by Tung et al. that used a multiomics approach to study differential physiological adaptations caused by intrinsic exercise capacity in male out bred ICR mice. After an exhaustive swimming test, mice were divided into three groups based on their exhaustive swimming times: 15 lowest exercise capacity mice or LEC group, 15 medium exercise capacity mice or MEC group, and 15 highest exercise capacity mice or HEC group. The analysis of the gut microbiota revealed that HEC mice had a greater microbial abundance and diversity than LEC mice, which suggests a high correlation with exercise capacity. In addition, the ratio of *Firmicutes/Bacteroidetes* was significantly higher in HEC mice than in LEC mice [137,138,139]. Recently, the potential role of gut microbiome manipulation on physical performance has been explored [140,141], but the hypothetical underlying mechanisms of a possible effect, are still unrevealed. In fact, Hsu et al. in 2015 were the first to investigate the association between intestinal bacteria and exercise performance in specific pathogen-free (SPF), germ-free (GF), and *Bacteroides fragilis* (BF) gnotobiotic mice. Given the modulating effects of gut microbiota on antioxidant enzyme activity and the potential link between enhanced antioxidant enzymes and exercise performance improvement [142], they examined antioxidant enzyme levels and endurance exercise after an exhaustive exercise challenge (endurance swimming test). The results showed decreased antioxidant enzyme activities and exercise performance in absence of microbiota (GF conditions), while monocolonization of GF mice with BF prevented the decline in endurance exercise time, suggesting that different microbiota status affects exercise performance by regulating antioxidant enzyme activity [140]. Also, Huang et al. in 2019 applied a gnotobiotic animal model to directly investigate the relationship of specific gut microbes and exercise physiology, showing a positive effect of the butyrogenic microbe *E. rectale* on exercise performance both with and without exercise training intervention, possibly due to improved bioavailability of energy [141]. Along with microbial composition, gut metabolic activity might contribute to physical performance, as indicated by studies that have observed an increase of biochemical pathways related to carbohydrate and amino acid metabolism in athletes’ microbiome compared with sedentary individuals [16,17,143]. Specifically, the gut microbiome produces SCFAs from dietary fiber fermentation, and increased levels of these fecal metabolites have been detected in response to physical activity [16,144]. Even though, normally in humans SCFAs contribute only by 1.2–10% to total energy during a western diet, compared to 57% of lowland gorillas [145,146,147], this percentage may influence energy availability during endurance performance, as reported in recent studies in murine models [143,148]. In particular, acetate, the dominant SCFA, appears to be the most important energy source in muscle [148], while propionate may be produced in skeletal muscles during anaerobic exercise by microbial lactate-utilizing species from systemic lactate and entering the gut lumen [143]. Furthermore, gut microbiota might also affect exercise performance by influencing skeletal muscle metabolism, function and fiber phenotype. Interestingly, Yan et al. found that germ-free (GF) mice replicate the fiber characteristics and lipid metabolic profile of the donor’s skeletal muscle after microbiota transplantation from obese or lean pigs [149]. Lahiri et al. compared the skeletal muscle of germ-free (GF) mice to skeletal muscle of pathogen-free (PF) mice, revealing that GF mice showed muscle atrophy, reduced muscle strength, decreased expression of IGF-1 and reduced transcription of genes associated with muscle growth and mitochondrial function. Transplanting the gut microbiota from PF mice into GF mice resulted in increased skeletal muscle mass, reduced muscle atrophy markers, improved oxidative metabolic capacity of the muscle, and elevated expression of neuromuscular junction assembly genes. Moreover, the administration of SCFAs to GF mice partly reversed skeletal muscle impairment [150]. Conversely, even though Nay et al. measured the endurance capacity decrease assessed by in vivo running tests and ex vivo muscle contractility tests in antibiotic treated healthy mice, they did not find any variations in muscle mass, fiber typology, or mitochondrial function. In their study, the decrease of skeletal muscle endurance was correlated with reduced glucose metabolism markers, such as expression of the SCFAs transporter GPR41, sodium-glucose cotransporter 1 (SGTL1) and muscle glycogen level, suggesting a correlation to the antibiotic-mediated gut microbiota depletion (21 days). Indeed, these effects were normalized after 10 days of natural reseeding, indicating that a glucose homeostasis improvement might contribute to ameliorate physical performance through gut microbial changes [59]. Figure 2 depicts the above reported studies in murine model.

## 6. Studies in Humans

### 6.1. Studies in Different Body Mass Index (BMI), Active and Sedentary Groups

Clinical studies indicate that exercise promotes a health-associated microbial community and increases metabolic functional potential, leading to the hypothesis of a possible link between gut microbiota, physical fitness, and wellness maintenance. To date, conclusive evidence on the effects of exercise on gut microbiota are lacking, as it seems that the observed shifts in microbial populations and metabolites occur as a result of an interplay of factors, including individual physical fitness status and/or specific nutrition regimens. Recently, Bressaet al al. conducted an observational study on 40 normal-weight premenopausal women divided in two groups, active and sedentary, demonstrating that physical activity performed at the minimum doses recommended by WHO (i.e., 3 days of exercise per week/ 30 min at a moderate intensity) but continuously, increased the abundance of the butyrate producers *Faecalibacterium prausnitzii* and *Roseburia hominis* [151], and of *Akkermansia muciniphila*, associated with a better body composition and improved metabolic health [152]. No changes were detected in the relative abundance of Bacteroidetes and Firmicutes, but a trend for a lower presence of the Bacteroidetes population in the active group was detected, consistent with findings obtained in professional athletes [153]. The microbiota diversity did not significantly differ between groups, but sedentary parameters (i.e., sedentary time and breaks) negatively correlated with microbiota richness [111]. Moreover, the following year Munukka et al. (2018) showed that a 6-week guided endurance exercise program (three training sessions/week in groups of 2–4 subjects performed as further described) modestly modified the composition and functions of gut microbiota among previously sedentary overweight women. More specifically, the exercise training increased the abundance of Verrucomirobia, a phylum that contains only a few species, and further *Akkermansia*, one of the major representatives of Verrucomicrobia. Moreover, exercise intervention decreased the abundance of Proteobacteria and an unidentified genus of Enterobacteriaceae, an increase of which has been reported in obesity and non-alcoholic steatohepatosis [119,154,155]. In the same year, a longitudinal study by Allen et al. examined the effects on gut microbiota in 32 previously sedentary normal weight or obese adults, providing a 6-week supervised endurance exercise program followed by a 6-week washout period. The results showed an increase in Faecalibacterium species and a decrease in *Bacteroides* species in normal weight subjects, while the opposite result was registered in obese individuals. Additionally, exercise increased fecal concentrations of SCFAs in lean, but not in obese participants, and, furthermore, this increase was transient and disappeared in the subsequent sedentary washout period, suggesting that exercise-induced alterations in gut microbiota depend on obesity status, and that the transient effect may be explained by the need of continuous training stimuli [144]. Accordingly, a prospective study by Cronin et al. showed that the gut microbial shifts observed in habitual exercisers and professional athletes might represent late responses to exercise or fitness. However, they did not identify a significant impact of 8 weeks of combined aerobic and resistance training on gut microbiome diversity and metabolic pathways in sedentary adult volunteers (predominantly overweight or obese), thus suggesting that this period length could not be enough to exert gut microbial changes [156]. The longest exercise intervention study was carried out by Kern et al. in 88 sedentary overweight or obese participants, divided in four arms (habitual living, active commuting by bike, leisure-time exercise of moderate intensity, or vigorous intensity) that completed a 6-month randomized controlled trial. Results showed that α-diversity increased in the vigorous intensity exercise group already after 3 months compared to controls, but associations between α-diversity and phenotypical outcomes such as cardiorespiratory fitness and fat mass were not observed. β-Diversity changed in all exercise groups compared with controls and, particularly, a decreased heterogeneity was detected in the vigorous intensity exercise group. No significant changes at the genus level were found. Finally, the inferred functional potential of the microbiota in the exercise groups was increased, primarily at 3 months [157].

### 6.2. Gut Microbiota as Function of Cardiorespiratory Fitness

Some studies have evaluated the gut microbiota composition as a function of cardiorespiratory fitness. The first study by Estaki et al. conducted on 39 healthy young adults with similar BMI and nutritional regimes, showed that gut microbial diversity (α-diversity) in healthy humans was associated with aerobic fitness as measured by peak oxygen uptake (VO_2_peak), while β-diversity analysis did not show distinct clustering of bacterial communities based on fitness (high, average, low) categories. However, a core set of gene-related functions rather than of bacterial taxa, was detected in subjects with high cardiorespiratory fitness. Additionally, a strong positive correlation was observed between VO_2_peak and fecal butyric acid, and an increased abundance of key butyrate-producing taxa (*Clostridiales*, *Roseburia*, *Lachnospiraceae*, and *Erysipelotrichaceae*) was detected amongst high physically fitted individuals [65]. These findings are consistent with those reported by Durket et al., who recently explored the relationship between cardiorespiratory fitness (maximal oxygen consumption, VO_2_max) and relative gut microbiota composition assessed by Firmicutes to Bacteroidetes ratio (F/B) in 37 healthy young adults. The results showed that VO_2_max was positively associated with an increase in F/B ratio, thus suggesting that exercise training may elicit favorable shifts in gut microbial composition in young healthy adults [158]. Conversely to these results, Cronin et al. found that the improvement in both cardiorespiratory fitness and body composition induced by 8 weeks of mixed aerobic and resistance exercise among overweight or obese healthy volunteers, were not dependent on a substantial alteration of the diversity of gut microbial populations [156]. Yang et al. conducted a study on 71 sedentary premenopausal women, mostly overweight or obese, divided into three groups based on the level of cardiorespiratory fitness (low, moderate and high) as assessed by the bicycle ergometer test [159]. The study demonstrated that the high fitness group had higher proportions of *Bacteroides* and lower *Eubacterium rectale Clostridium coccoides* (EreC) than the low fitness group as also previously shown by other studies demonstrating that EreC group is associated with obesity and related to metabolic disorders [115,119,160,161]. In this study, VO_2_max was inversely associated with EreC, however, after adjusting for fat percentage, this association disappeared [159] as similarly demonstrated in 2019 by Morita and colleagues. They showed that aerobic exercise training targeting an increase of the time spent in brisk walking increased the relative abundance of intestinal *Bacteroides*, whereas trunk muscle training (TM) did not change the composition of the intestinal microbiota in subjects within the TM group [162]. Whether cardiorespiratory fitness plays a role in altering gut microbiota is still unclear, as the underlying mechanisms by which improvements in fitness profile might be associated with microbiota are yet to be fully understood. Moreover, as discussed by Estaki et al., other components of fitness such as anaerobic capacity and resistance muscle training may also influence microbial community composition [65]. Although diversity of results is evident from human studies, a link between exercise and both taxonomic and functional alterations of gut microbial community has been observed. As reported by Mitchell et al. in a systematic review of 2019, the greatest difficulties in studying the relationship between exercise and gut microbiota included the ability to distinguish the effects of diet from those induced by exercise, and the different outcomes of longer and/or higher intensity training protocols, plus the identification of specific gut microbial responses among physically active or inactive, in either normal weight or overweight/obese individuals [131].

### 6.3. Studies in Athletes

The studies conducted on physically active and sedentary subjects indicate a role of exercise in gut microbiome modification, therefore, given the remarkable physiological and metabolic adaptations of athletes, it is highly expectable to observe clear differences between microbiota characteristics in athletes and sedentary individuals. Accordingly, several studies reported a higher abundance of health-promoting bacterial species, increased microbial diversity, and functional/metabolic potential in gut microbiota of athlete populations [16,17,35].

#### 6.3.1. Endurance Sport Studies

Potential alteration in gut microbiota composition and microbial metabolic profile in endurance athletes was first evaluated by Zhao and colleagues, who performed an untargeted metabolomics methodology and 16S ribosomal DNA (rDNA) sequencing analysis in 20 amateur half-marathon runners. More specifically, 40 fecal samples were collected before and after a half-marathon race and during the two sample time periods each volunteer was given the same kind of food. The study did not reveal any significant differences in α-diversity, but more specific bacterial taxa ranking from phylum to species levels were detected after than before the half-marathon race, thus indicating that running potentially increased the diversity of the gut microbiota [163]. Interestingly, increased species richness was observed after running in the Coriobacteriaceae family, that appear to be involved in the metabolism of bile salts and steroids as well as in the activation of dietary polyphenols in the human gut [164]; this increase was shown to correlate with 15 differential metabolites, suggesting that the metabolism of Coriobacteriaceae might be the potential mechanism underlying the role of exercise in preventing disease and improving health outcomes. Functional prediction to determine potential functions of the gut microbiota, that may be altered by intensive running, showed that “cell motility” function of gut microbiota was significantly induced after running, while “energy production and conversion” was repressed. Finally, correlation analysis indicated that the observed differences in gut microbiome might have been the shared outcome of running and dietary intervention. Additionally, significant correlations between food intake and gut microbiota composition were found, with fat and energy intake as major factors that could alter microbial communities in a rapid manner [163]. Moreover, Scheiman et al. carried out a study in the category of marathon runners, by examining gut microbiota of 15 athletes that performed the Boston Marathon in 2015, along with 10 sedentary controls, to identify gut bacteria associated with athletic performance and recovery states. They conducted 16S rDNA sequencing on approximately daily stool samples collected up to one week before and one week after marathon day, showing that there was a significant difference in the relative abundance of *Veillonella* genus between samples collected before and after exercise. Moreover, the prevalence of *Veillonella* appeared higher among marathon runners than non-runners, but this difference was not statistically significant. These data suggest that systemic lactate produced during exercise could be accessible to the microbiome and converted to SCFAs that improve athletic performance. So *Veillonella* could have a symbiotic relationship in the human microbiome [143]. Further studies by Keohane et al. focused on the response of the gut microbiota to prolonged intense exercise in previously active individuals, by exploring the changes in microbial diversity, abundance, and metabolic capacity in four well-trained ultra-endurance male athletes that performed prolonged, high-intensity trans-oceanic rowing. They collected serial stool samples from athletes before, during, and after a continuous, unsupported 33-day, 5000 km transoceanic rowing race and studied microbial community structure and relevant functional gene profiles by whole-genome shotgun sequencing analysis. Dietary data were recorded by a validated food frequency questionnaire, and body composition analysis and cardiorespiratory testing were performed. The results indicated an increase of α-diversity throughout the ultra-endurance race (except in one rower, who required antibiotic treatment before midrace), that occurred independently of any change in cardiorespiratory fitness, with VO2max similar pre- and postrace. Variations in taxonomic composition comprised increased abundance of butyrate producing species and species associated with improved metabolic health, including high insulin sensitivity. At the functional metabolic level, an increase of S-adenosyl methionine (SAMe), medium and long chain fatty acids and specific amino acids (i.e., L-isoleucine and L-lysine) biosynthesis pathways were observed. Interestingly, many of the adaptions in microbial community structure and metaproteomics persisted up to 3 months follow-up, suggesting that both gut microbial diversity and metabolic potential are positively influenced by prolonged and intense exercise, and these adaptions may play a compensatory role in controlling the physiological stress associated with sustained exertion as well as negating the deleterious consequences of endurance exercise. However, the different diet followed during the rowing race with respect to the pre-race period, could also have influenced the changes observed in gut microbial community structure and function [165]. A recent interesting study by Hampton-Marcell et al. has focused on the temporal dynamics of the association between exercise and gut microbial community alterations, by exploring microbial shifts in response to short-term changes in training volume among Division I NCAA collegiate swimmers. Fecal sample collection was conducted during peak training through swimmers’ in-season taper in 2016 (*n* = 9) and 2017 (*n* = 7), capturing a systematic reduction in training volume (measured as swimming distance) near the conclusion of the athletic season. The 16S rRNA V4 amplicon sequencing showed a reduction in training volume during the study period, that coincided with a significant decrease in overall microbial diversity, microbial community structural similarity [166].

#### 6.3.2. Team Sports Studies

The first study on athletes was carried out by Clarke et al. in 2014 in which male elite professional rugby players and two control groups, one matched for athlete physical size (BMI > 28), and another matched for age and gender (BMI ≤ 25) were evaluated. The analysis of gut microbiota revealed that the α-diversity of the elite athlete microbiota was significantly higher, with 22 phyla detecting with respect to 11 phyla in BMI ≤ 25 and 9 phyla in BMI > 28 control groups, and this enhanced diversity correlated with protein consumption and plasma creatine kinase. In particular, the greater diversity was among the Firmicutes phylum, while Bacteroidetes taxon was less abundant. Another interesting data was that both athletes and the low BMI groups had significantly higher proportions of the genus *Akkermansia*, whose abundance inversely correlates with obesity and associated metabolic disorders in mice and humans [35,167,168]. In 2018, Barton et al. reexamined the microbiome in these participants (both athletes and controls) by metabolic phenotyping and functional metagenomic analysis, to further investigate whether the microbial differences did correspond to distinctive functional/metabolic features. They found that differences in fecal microbiota between athletes and sedentary controls were even more pronounced at the functional/metabolic level than at the compositional level as previously reported. In particular, athletes had an increased abundance of pathways that could be exploited by the host for potential health benefits (i.e., biosynthesis of organic cofactors and antibiotics, as well as carbohydrate degradation and secondary metabolite metabolism), and increased levels of fecal SCFAs relative to controls [17].

#### 6.3.3. Studies across Different Levels of Athletes

Petersen et al. examined the gut microbiota of 22 professional and 11 amateur level competitive cyclists by metagenomic whole genome shotgun sequencing and RNA sequencing analyses, describing a significant correlation between time reported exercising during an average week and the abundance of the genus *Prevotella*, independently of the athletes’ category (professional or amateur). *Prevotella* abundance has been correlated to the number of average kilocalories consumed per day and diets high in complex carbohydrates (including high dietary fiber from various sources such as fruits and vegetables), egg food items, and high vitamins and minerals [169,170,171]. Furthermore, metatranscriptome analysis revealed the up regulation of branched chain amino acid (BCAA) biosynthesis when there was an increase in *Prevotella* transcripts. Of interest, another difference between the metagenome and metatranscriptome analysis level, was the significant increase in transcriptional activity of *Methanobrevibacter smithii* in the number of professional cyclists in comparison to amateur cyclists, and this archaeon showed an up regulation of those genes involved in the production of methane. Finally, this upregulation of methane metabolism was associated with a similar increase of energy and carbohydrate metabolism pathways [16]. A further study by Liang et al. examined the differentiation of the gut microbiota characteristics in 31 professional routine martial arts athletes divided into two groups according to their qualification (higher-level, H group and lower-level, L group). They found that higher-level athletes had significantly higher gut microbial diversity and richness (α-diversity index) than lower-level athletes. Moreover, the higher-level athletes also had a different gut microbial structure (β-diversity index) than the lower-level athletes. Among the taxa that were significantly different between the two groups, the genera *Parabacteroides* and *Phascolarctobacterium* were higher in the H group than in the L group, and *Megasphaera* was lower. Of interest, the abundance of *Parabacteroides*, which is closely associated with exercise and cardiac function, and negatively with metabolic disorders [172,173,174] was remarkably correlated with exercise load, while the other higher abundant genus *Phascolarctobacterium* has been reported to produce SCFAs including propionate and acetate [175]. Conversely, the abundant genus *Megasphaerain* the lower-level athletes (which has been suggested to be more abundant in chronic inflammatory diseases, such as nonalcoholic steatohepatitis and genital tract inflammation), has the potential to produce high levels of LPS [176,177]. Furthermore, the analysis of the functional prediction revealed that histidine and carbohydrate metabolism pathways were markedly overrepresented in the gut microbiota of higher-level athletes [178].

#### 6.3.4. Studies across Different Sport Classifications

**Figure 3:**
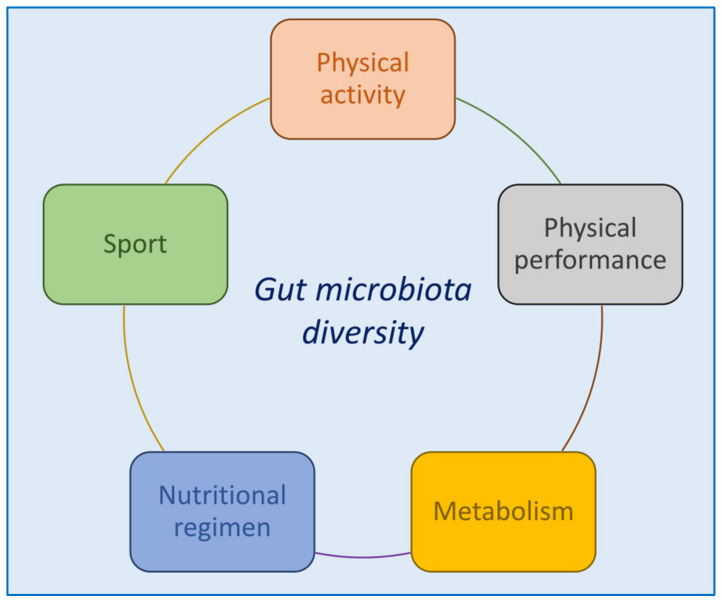
Schematic diagram depicting the factors that contribute to modify the gut microbiota in humans.

A recent study by O’Donovan et al. investigated the impact of different training types on gut microbiome and metabolomes, by studying 37 elite Irish athletes who competed across 16 different sports classified into broader sport classification group (SCGs) based on the static and dynamic components of the sport. Differences were observed in microbial composition between SCGs. Individual variability was detected across athletes, with clustering of the majority of samples driven by the relative abundances of five species, namely, *Eubacteriumrectale*, *Polynucleobacternecessarius*, *Faecalibacteriumprausnitzii*, *Bacteroides vulgatus* and *Gordonibactermassiliensis*. The species composition changes may be the result of the variance in demand across different types of sports, including duration of activity and training modes. Of interest, no significant differences in dietary intake were found between any nutrients or food groups based on SCGs, gender, or location of sample collection after correction for multiple comparisons [179]. Jang et al. investigated the long-term effects of a specific exercise type and athletes’ diets on gut microbiota, by comparing microbial characteristics, dietary intake, and body composition of male bodybuilders (*n* = 15), male elite distance runners (*n* = 15) and healthy men (*n* = 15) without regular exercise habits that served as controls. They found that the type of exercise training and the diet pattern associated with specific sports (high fat, high protein and low carbohydrate/low fiber diet for bodybuilders, and low-carbohydrate and low-dietary fiber diet for distance runners) did not make a difference in the β-diversity of gut microbiota, but they did affect the relative abundance of certain intestinal microbes. In particular, at the genus level, bodybuilders showed a reduction of relative abundance of *Bifidobacterium* and an increase of *Sutterella*; *Bifidobacterium* was negatively correlated with fat intake, whereas *Sutterella* (associated with both high-fat and low-fiber diet) was positively correlated with fat intake [180]. Moreover, in bodybuilders, acetate-producing bacteria (i.e., *Blautiawexlerae*, *Bifidobacterium adolescentis group*, and *Bifidobacterium longum group*) and lactate-producing bacteria (i.e., *Lactobacillus sakeigroup*) diminished, thus influencing the substrate supply of butyrate-producing bacteria, such as *Eubacterium hallii*. In distance runners, gut microbiota diversity tended to decrease as protein intake increased. Taken together, these results suggest that high-protein diets may have a negative impact on gut microbiota diversity for athletes performing endurance sports who consume low carbohydrates and low dietary fiber, while athletes performing resistance sports, who carry out a high protein, low carbohydrate and high fat diet, demonstrate a decrease in short chain fatty acid-producing commensal bacteria [181]. The heterogeneous findings in athletes’ studies highlight how gut microbial composition and functional profile in this population likely derives from a combination of intrinsic and extrinsic factors that comprise the host characteristics (i.e., metabolic features and physical fitness level), and environmental factors, including an overall healthy lifestyle, in particular dietary habits. Athletes often adhere to strict dietary regimens to support training and performance, particularly when they perform extreme exercise, such as higher calory and protein intake, high carbohydrate (both complex and simple) consumption, and the use of vitamin supplements, and dietary differences represent a confounding variable in the interpretation of the independent role of exercise on gut microbial changes [15]. In fact, long-term dietary patterns, particularly the consumption of mostly animal protein and fat (*Bacteroides*) or mostly plant-derived carbohydrate (*Prevotella*) diet, are strongly associated with enterotype partitioning, due to the high level of resilience of the bacterial community [170]. While some studies observed a negative impact of high-protein diets on gut microbiota diversity as well as on the amount of SCFAs-producing commensal bacteria in athletes [181], other studies found that high protein consumption increased microbial diversity [15], but these inconsistencies may depend on the different dietary fiber intake among athletes populations [182], microbial proteolytic fermentation (with subsequent production of compounds that can negatively impact barrier function and host physiological response) [183], occurs in specific situations, such as when the intake of protein is elevated [184] and when low-fiber-low-resistant starches are provided [185]. Moreover, the microbiota-accessible carbohydrates (MAC) found within specific foods have the ability to suppress protein fermentation by lowering pH in the distal gut and decreasing the requirement of amino acids as an energy source for microorganisms [183]. Other important confounding factors are attributable to the numerous differences in diverse athlete groups that include exercise characteristics (i.e., modality, intensity, frequency, etc.), competition level (amateur or professional), training history and features (i.e., on-season and off-season programmes, open or closed training environment). Specific sport disciplines and subsequent exercise modalities and intensity might also influence gut microbiota responses by determining intrinsic adaptations in the gastrointestinal (GI) tract, such as transit time and gut pH modifications. These adaptive mechanisms may contribute to the building of an environmental setting that favors richer microbial diversity and metabolic functions [64,65], but they also may hinder these effects. For example, in prolonged excessive exercise, as frequently occurs in endurance sports, increased intestinal permeability with subsequent endotoxemia may occur as a consequence of splanchnic hypoperfusion and ischemia and subsequent reperfusion [49,123,127]. The combination of endotoxemia and undersupply of blood nutrients, water and oxygen to the GI tract, results in detrimental symptoms such as nausea, cramping, vomiting, and diarrhea [186]. Therefore, studies on the possible mechanisms by which gut microbiota may improve intestinal barrier function in athletes are further required [49,127]. Figure 3 summarizes the above reported studies on humans.

## 7. Conclusions and Future Prospective

Physical exercise performed at the doses recommended by the World Health Organization (WHO) improves fitness and quality of life throughout different mechanisms likely involving modification of gut microbiota. The current body of literature indicates that regular exercise increases gut microbial diversity and promotes the growth of symbiotic bacteria populations and functional pathways, leading to a healthier status and possibly, also enhancing physical performance in athletes. The effects of exercise on the gut microbiome seem to be influenced by several factors, including the physiological state of the individual, as suggested by a richer microbiota in physically active subjects, variables related to exercise such as intensity and timing, likely linked to both positive and negative outcomes, and long-term dietary habits, particularly protein and carbohydrate (fiber) intake. A difficult task to reach is to untie the contribution of the different extrinsic and intrinsic determinants impacting on the microbial community. The interaction between physical activity and gut microbiota should be further characterized by taking into account the confounding variables, by setting standardized methods in study design (i.e., fecal sampling protocols, sequencing and analytical techniques, statistical analysis), by controlling calories intake, but also nutrients quality and composition, and by fixing exercise parameters (i.e., acute or chronic exercise, cardio and/or resistance training, duration and frequency). Furthermore, the underlying mechanisms by which exercise exerts its effects on the intestinal microbiota should be elucidated by integrating taxonomy studies with metagenomics, metatranscriptomics, metaproteomics and metabolomics. Functional diversity may be the main determinant of a properly operating microbial environment [187] and, consequently, of health host. Another aspect to further investigate regards the gut microbial sampling methodology, as it has been suggested that the fecal compartment composition may be not reliable in capturing the true complexity of the gut microbiota (both under or over- representing bacteria genera and species), without a clear distinction between the mucus layer and the lumen residing microbial populations and metabolites [35,36,37]. The available data on athletes suggest that this category presents distinct microbial features from those of sedentary individuals, characterized by amplified microbial diversity, and higher abundance of health-promoting bacterial associated with improved fitness status and overall health [15,16,17]. Gut microbial community changes and function occur as result of the interplay of exercise and dietary habits and might contribute to improve athletic performance and reduce recovery time during training, but further studies are required to enlighten the microbiota and exercise relationship and to gain novel opportunities, such as the ability to predict responses to interventions and athletic performance. Meta-omics data combined with computational strategies such as interpretable machine learning (ML) approaches may play a key role in revealing how microorganisms interact with each other and with their host to identify different healthy microbiota scenarios relevant for human health and, also, performance improvement. In fact, ML can provide profound insights on how an athlete’s physiology is influenced by several different factors. For instance, ML models could predict the athlete’s exercise responsiveness in terms of glucose homeostasis and insulin sensitivity [172] or in terms of biomarker signature of aerobic fitness [188]; such predictions might be used to provide a customized lifestyle recommendation for modulating an individual’s microbiota and consequently improving an athlete’s responsiveness to exercise, as well as to specific nutrients or supplements. Finally, whereas in sports these technologies may be used to maintain or improve performance, they might represent a novel tool for improving public health by optimizing diet and personal lifestyle [18]. In fact, given the remarkable physiology and metabolism of athletes, this population may serve as a crucial model in the exercise-gut microbiota research field, to transfer and apply knowledge to the entire health in larger communities [64]. Further studies on the interaction between exercise and microbiota should target the potential health benefits in older individuals, as gut microbial resilience after the age of 65 is generally reduced, and the overall composition is more vulnerable to lifestyle changes, drug treatment and diseases, leading to decreased species richness and enhanced interindividual variability. Moreover, gut microbiota biodiversity seems to inversely correlate with physical function and institutionalization of older individuals. The reduction of physical performance and muscle strength are the main features of frail individuals with sarcopenia, as these age-related conditions often coexist. Notably, gut dysbiosis may contribute to several metabolic disorders involved in sarcopenia and frailty, such as enhanced protein catabolism, pro-inflammatory cytokine production, decreased pro-anabolic mediators’ synthesis and reduced insulin sensitivity, with some of these alterations linked to gut mucosa dysfunction and increased permeability. Moreover, microbial disequilibrium could also be associated with reduced survival in older individuals with frailty or disability [189]. Since exercise, together with proper nutrition [190], is the most important tool for the prevention and treatment of these high prevalent geriatric conditions [191], longitudinal studies are needed to verify whether these effects may be mediated by recovery of health-related gut microbiota features along with body composition improvement in sarcopenic and frail subjects. Lastly, another opportunity that could be considered to extend knowledge on the exercise-microbiome connection consists in assessing the possible effects of exercise-induced gut microbiota alterations on skeletal muscle parameters, including muscle structure, mass, strength and function, and muscle metabolism that ultimately affect both skeletal muscle health and physical performance. In fact, several diet-derived compounds produced or modified by gut microbes can enter the systemic circulation and ultimately influence skeletal muscle cells, such as SCFAs, amino acids, secondary bile acids, polyphenols and vitamins [192]. To date, few studies, most on animal and gnotobiotic models, have been conducted, some of these suggesting a role of microbiota composition and metabolites in skeletal muscle homeostasis and functional capacity [59,140,141,143,148,150], therefore, future research in humans is needed to clarify clinical relevance in this field. In recent years, studies in animals and humans have confirmed a positive role of exercise in shaping a healthy gut microbiota, possibly contributing to enhanced human health and, also, physical performance. On the other hand, a condition of gut dysbiosis may negatively affect muscle mass and function, by promoting intestinal permeability, systemic inflammation, reduced nutrient availability, and decreased protein synthesis [193]. Novel intervention studies in healthy athletes and sedentary individuals or older populations could represent a step forward for the recognition of specific microbial taxa, pivotal active genes and related metabolic pathways, together with bioactive, hormone-like microbial metabolites involved in the gut-muscle axis, to further understand whether exercise might be a positive modulator of this complex cross-talk. 

## Figures and Tables

**Table 1:** Health-related functions of gut microbiota.

Metabolic-Endocrine Functions	References	Protective and Structural Functions	References
Production of Short Chain Fatty Acids (SCFAs)	[30,31,32,33,34]	Secretion of mucus and antimicrobial factors	[35,36,37]
Biosynthesis and absorption of nutrients (i.e., salts/water absorption, carbohydrate fermentation, vitamins and amino acids production)	[38]	Prevention of pathogenic colonization by competition for nutrients and attachment sites and antimicrobial activity	[39,40,41,42]
Bio-transformation of bile acids	[43,44]	Influence of innate and adaptive immune system and functions	[45,46,47]
Production of local neurotransmitters such as nitric oxide (NO), γ-aminobutyric acid (GABA) and monoamines (noradrenaline, dopamine, serotonin)	[48,49,50,51,52]	Regulation of inflammatory cytokines production	[53,54,55]
Activation of protein kinases	[56]	Modulation of tight junctions and intestinal permeability	[57,58]
Modulation of mitochondrial biogenesis	[59]	Promotion of epithelial cell growth and differentiation	[39,40]
Improvement of myofibers efficiency and protection of muscle protein catabolism	[60,61]	Micro-vascularization of intestinal villi and development of the crypts	[62,63,64,65]
Maintenance of glucose homeostasis and promotion of insulin sensitivity	[15,66,67]		
Regulation of host adiposity, leptin production and body weight	[15,68,69,70]		
Regulation of food intake and appetite	[66]		
Metabolism of drugs and xenobiotics	[39,40,41,42]		
Differentiation of enteroendocrine cells	[71]		

**Table 2:** Endogenous and exogenous determinants of gut microbiota community.

Determinants of Gut Microbiota	References
Host genetics and physiopathology	[36,97]
Age	[36]
Gender	[98]
Geographic origin	[99,100]
Pregnancy	[101]
Type of birth (natural or caesarian)	[102]
Method of infant feeding (breastfeeding or infant formula)	[103]
Dietary habits	[105,106,107,108,109]
Physical exercise and individual fitness status	[11,13,14,43,62,110,111]
Antibiotic and other drugs intake	[104]
Stress	[104]

## References

[1] Owen N., Sparling P.B., Healy G.N., Dunstan D.W., Matthews C.E. (2010). Sedentary behavior: Emerging evidence for a new health risk. Mayo Clin. Proc..

[2] Fiuza-Luces C., Garatachea N., Berger N.A., Lucia A. (2013). Exercise is the real polypill. Physiology.

[3] Gonzalez-Freire M., de Cabo R., Studenski S.A., Ferrucci L. (2014). The neuromuscular junction: Aging at the crossroad between nerves and muscle. Front. Aging Neurosci..

[4] MSilverman N., Deuster P.A. (2014). Biological mechanisms underlying the role of physical fitness in health and resilience. Interface Focus.

[5] Hawley J.A., Hargreaves M., Joyner M.J., Zierath J.R. (2014). Integrative biology of exercise. Cell.

[6] Hughes R.L. (2020). A Review of the Role of the Gut Microbiome in Personalized Sports Nutrition. Front. Nutr..

[7] Ley R.E., Bäckhed F., Turnbaugh P., Lozupone C.A., Knight R.D., Gordon J.I. (2005). Obesity alters gut microbial ecology. Proc. Natl. Acad. Sci. USA.

[8] Boulangé C.L., Neves A.L., Chilloux J., Nicholson J.K., Dumas M.E. (2016). Impact of the gut microbiota on inflammation, obesity, and metabolic disease. Genome Med..

[9] Cryan J.F., O’Mahony S.M. (2011). The microbiome-gut-brain axis: From bowel to behavior. Neurogastroenterol. Motil..

[10] Dalton A., Mermier C., Zuhl M. (2019). Exercise influence on the microbiome–gut–brain axis. Gut Microbes.

[11] Petriz B.A., Castro A.P., Almeida J.A., Gomes C.P., Fernandes G.R., Kruger R.H., Pereira R.W., Franco O.L. (2014). Exercise induction of gut microbiota modifications in obese, non-obese and hypertensive rats. BMC Genom..

[12] Evans C.C., LePard K.J., Kwak J.W., Stancukas M.C., Laskowski S., Dougherty J., Moulton L., Glawe A., Wang Y., Leone V. (2014). Exercise Prevents Weight Gain and Alters the Gut Microbiota in a Mouse Model of High Fat Diet-Induced Obesity. PLoS ONE.

[13] Liu Y., Wang Y., Ni Y., Cheung C.K.Y., Lam K.S.L., Wang Y., Xia Z., Ye D., Guo J., Tse M.A. (2020). Gut Microbiome Fermentation Determines the Efficacy of Exercise for Diabetes Prevention. Cell Metab..

[14] Motiani K.K., Collado M.C., Eskelinen J.-J., Virtanen K.A., Löyttyniemi E., Salminen S., Nuutila P., Kalliokoski K.K., Hannukainen J.C. (2020). Exercise training modulates gut microbiota profile and improves endotoxemia. Med. Sci. Sports Exerc..

[15] Clarke S., Murphy E.F., O’Sullivan O., Lucey A., Humphreys M., Hogan A., Hayes P., O’Reilly M., Jeffery I., Wood-Martin R. (2014). Exercise and associated dietary extremes impact on gut microbial diversity. Gut.

[16] Petersen L.M., Bautista E.J., Nguyen H., Hanson B.M., Chen L., Lek S.H., Sodergren E., Weinstock G.M. (2017). Community characteristics of the gut microbiomes of competitive cyclists. Microbiome.

[17] Barton W., Penney N.C., Cronin O., Garcia-Perez I., Molloy M.G., Holmes E., Shanahan F., Cotter P.D., O’Sullivan O. (2018). The microbiome of professional athletes differs from that of more sedentary subjects in composition and particularly at the functional metabolic level. Gut.

[18] Mancin L., Rollo I., Mota J.F., Piccini F., Carletti M., Susto G.A., Valle G., Paoli A. (2021). Optimizing Microbiota Profiles for Athletes. Exerc. Sport Sci. Rev..

[19] Sender R., Fuchs S., Milo R. (2016). Are We Really Vastly Outnumbered? Revisiting the Ratio of Bacterial to Host Cells in Humans. Cell.

[20] Qin J., Li R., Raes J., Arumugam M., Burgdorf K., Manichanh C., Nielsen T., Pons N., Levenez F., Yamada T. (2010). A human gut microbial gene catalogue established by metagenomic sequencing. Nature.

[21] Ley R.E., Peterson D.A., Gordon J.I. (2006). Ecological and Evolutionary Forces Shaping Microbial Diversity in the Human Intestine. Cell.

[22] Ringel-Kulka T., Palsson O.S., Maier D., Carroll I., Galanko J.A., Leyer G., Ringel Y. (2011). Probiotic Bacteria Lactobacillus acidophilus NCFM and Bifidobacterium lactis Bi-07 Versus Placebo for the Symptoms of Bloating in Patients with Functional Bowel Disorders: A double-blind study. J. Clin. Gastroenterol..

[23] Mariat D., Firmesse O., Levenez F., Guimaraes V.D., Sokol H., Dore J., Corthier G., Furet J.-P. (2009). The Firmicutes/Bacteroidetes ratio of the human microbiota changes with age. BMC Microbiol..

[24] Alonso V.R., Guarner F. (2013). Linking the gut microbiota to human health. Br. J. Nutr..

[25] Fanning S., Proos S., Jordan K., Srikumar S. (2017). A Review on the Applications of Next Generation Sequencing Technologies as Applied to Food-Related Microbiome Studies. Front. Microbiol..

[26] Srinivasan R., Karaoz U., Volegova M., MacKichan J., Kato-Maeda M., Miller S., Nadarajan R., Brodie E., Lynch S.V. (2015). Use of 16S rRNA Gene for Identification of a Broad Range of Clinically Relevant Bacterial Pathogens. PLoS ONE.

[27] Riesenfeld C.S., Schloss P.D., Handelsman J. (2004). Metagenomics: Genomic Analysis of Microbial Communities. Annu. Rev. Genet..

[28] Almeida A., Mitchell A., Boland M., Forster S., Gloor G., Tarkowska A., Lawley T.D., Finn R.D. (2019). A new genomic blueprint of the human gut microbiota. Nature.

[29] Gallè F., Valeriani F., Cattaruzza M. (2019). Exploring the association between physical activity and gut microbiota composition: A review of current evidence. Ann. Ig..

[30] Brown A.J., Goldsworthy S.M., Barnes A.A., Eilert M.M., Tcheang L., Daniels D., Muir A.I., Wigglesworth M.J., Kinghorn I., Fraser N.J. (2003). The Orphan G Protein-coupled Receptors GPR41 and GPR43 Are Activated by Propionate and Other Short Chain Carboxylic Acids. J. Biol. Chem..

[31] Le Poul E., Loison C., Struyf S., Springael J.-Y., Lannoy V., Decobecq M.-E., Brezillon S., Dupriez V., Vassart G., Van Damme J. (2003). Functional Characterization of Human Receptors for Short Chain Fatty Acids and Their Role in Polymorphonuclear Cell Activation. J. Biol. Chem..

[32] Ekimura I., Einoue D., Ehirano K., Etsujimoto G. (2014). The SCFA Receptor GPR43 and Energy Metabolism. Front. Endocrinol..

[33] Den Besten G., van Eunen K., Groen A.K., Venema K., Reijngoud D.-J., Bakker B.M. (2013). The role of short-chain fatty acids in the interplay between diet, gut microbiota, and host energy metabolism. J. Lipid Res..

[34] Besten G.D., Lange K., Havinga R., Van Dijk T.H., Gerding A., Van Eunen K., Muller M., Groen A.K., Hooiveld G., Bakker B. (2013). Gut-derived short-chain fatty acids are vividly assimilated into host carbohydrates and lipids. Am. J. Physiol. Liver Physiol..

[35] Clarke G., Stilling R., Kennedy P.J., Stanton C., Cryan J., Dinan T.G. (2014). Minireview: Gut Microbiota: The Neglected Endocrine Organ. Mol. Endocrinol..

[36] O’Toole P. (2012). Changes in the intestinal microbiota from adulthood through to old age. Clin. Microbiol. Infect..

[37] Zmora N., Zilberman-Schapira G., Suez J., Mor U., Dori-Bachash M., Bashiardes S., Kotler E., Zur M., Regev-Lehavi D., Brik R.B.-Z. (2018). Personalized Gut Mucosal Colonization Resistance to Empiric Probiotics Is Associated with Unique Host and Microbiome Features. Cell.

[38] Rafiki A., Boulland J.-L., Halestrap A., Ottersen O., Bergersen L. (2003). Highly differential expression of the monocarboxylate transporters MCT2 and MCT4 in the developing rat brain. Neuroscience.

[39] Egrenham S., Clarke G., Cryan J.F., Dinan T.G. (2011). Brain-Gut-Microbe Communication in Health and Disease. Front. Physiol..

[40] Bäckhed F., Ley R.E., Sonnenburg J.L., Peterson D.A., Gordon J.I. (2005). Host-Bacterial Mutualism in the Human Intestine. Science.

[41] Li H., He J., Jiaojiao H. (2016). The influence of gut microbiota on drug metabolism and toxicity. Expert Opin. Drug Metab. Toxicol..

[42] Noh K., Kang Y.R., Nepal M.R., Shakya R., Kang M.J., Kang W., Lee S., Jeong H.G., Jeong T.C. (2017). Impact of gut microbiota on drug metabolism: An update for safe and effective use of drugs. Arch. Pharmacal Res..

[43] Evans J.M., Morris L.S., Marchesi J. (2013). The gut microbiome: The role of a virtual organ in the endocrinology of the host. J. Endocrinol..

[44] Tremaroli V., Bäckhed F. (2012). Functional interactions between the gut microbiota and host metabolism. Nature.

[45] Naccache P.H., Faucher N., Caon A.C., McColl S.R. (1988). Propionic acid-induced calcium mobilization in human neutrophils. J. Cell. Physiol..

[46] Nakao S., Fujii A., Niederman R. (1992). Alteration of cytoplasmic Ca^2+^ in resting and stimulated human neutrophils by short-chain carboxylic acids at neutral pH. Infect. Immun..

[47] Nakao S., Moriya Y., Furuyama S., Niederman R., Sugiya H. (1998). Propionic acid stimulates superoxide generation in human neutrophils. Cell Biol. Int..

[48] DeCastro M., Nankova B.B., Shah P., Patel P., Mally P.V., Mishra R., La Gamma E.F. (2005). Short chain fatty acids regulate tyrosine hydroxylase gene expression through a cAMP-dependent signaling pathway. Mol. Brain Res..

[49] Clark A., Mach N. (2016). Exercise-induced stress behavior, gut-microbiota-brain axis and diet: A systematic review for athletes. J. Int. Soc. Sports Nutr..

[50] Ji X.-B., Hollocher T.C. (1988). Reduction of nitrite to nitric oxide by enteric bacteria. Biochem. Biophys. Res. Commun..

[51] Salzman A.L. (1995). Nitric oxide in the gut. New Horiz..

[52] Purvis D., Gonsalves S., Deuster P.A. (2010). Physiological and Psychological Fatigue in Extreme Conditions: Overtraining and Elite Athletes. PM&R.

[53] Macia L., Thorburn A.N., Binge L.C., Mariño E., Rogers K.E., Maslowski K., Vieira A., Kranich J., Mackay C.R. (2011). Microbial influences on epithelial integrity and immune function as a basis for inflammatory diseases. Immunol. Rev..

[54] Maslowski K.M., Vieira A.T., Ng A., Kranich J., Sierro F., Yu D., Schilter H.C., Rolph M.S., Mackay F., Artis D. (2009). Regulation of inflammatory responses by gut microbiota and chemoattractant receptor GPR43. Nature.

[55] Furusawa Y., Obata Y., Fukuda S., Endo T.A., Nakato G., Takahashi D., Nakanishi Y., Uetake C., Kato K., Kato T. (2013). Commensal microbe-derived butyrate induces the differentiation of colonic regulatory T cells. Nature.

[56] Dukes A., Davis C., El Refaey M., Upadhyay S., Mork S., Arounleut P., Johnson M.H., Hill W.D., Isales C.M., Hamrick M.W. (2015). The aromatic amino acid tryptophan stimulates skeletal muscle IGF1/p70s6k/mTor signaling in vivo and the expression of myogenic genes in vitro. Nutrition.

[57] Chen J., Guo Y., Gui Y., Xu D. (2018). Physical exercise, gut, gut microbiota, and atherosclerotic cardiovascular diseases. Lipids Health Dis..

[58] Karl J.P., Margolis L.M., Murphy N.E., Carrigan C.T., Castellani J., Madslien E.H., Teien H., Martini S., Montain S.J., Pasiakos S.M. (2017). Military training elicits marked increases in plasma metabolomic signatures of energy metabolism, lipolysis, fatty acid oxidation, and ketogenesis. Physiol. Rep..

[59] Nay K., Jollet M., Goustard B., Baati N., Vernus B., Pontones M., Lefeuvre-Orfila L., Bendavid C., Rué O., Mariadassou M. (2019). Gut bacteria are critical for optimal muscle function: A potential link with glucose homeostasis. Am. J. Physiol. Metab..

[60] Den Besten G., Gerding A., Van Dijk T.H., Ciapaite J., Bleeker A., Van Eunen K., Havinga R., Groen A.K., Reijngoud D.-J., Bakker B.M. (2015). Protection against the Metabolic Syndrome by Guar Gum-Derived Short-Chain Fatty Acids Depends on Peroxisome Proliferator-Activated Receptor γ and Glucagon-Like Peptide-1. PLoS ONE.

[61] Walsh M.E., Bhattacharya A., Sataranatarajan K., Qaisar R., Sloane L.B., Rahman M.M., Kinter M., Van Remmen H. (2015). The histone deacetylase inhibitor butyrate improves metabolism and reduces muscle atrophy during aging. Aging Cell.

[62] Matsumoto M., Inoue R., Tsukahara T., Ushida K., Chiji H., Matsubara N., Hara H. (2008). Voluntary Running Exercise Alters Microbiota Composition and Increases n-Butyrate Concentration in the Rat Cecum. Biosci. Biotechnol. Biochem..

[63] Leonel A.J., Alvarez-Leite J. (2012). Butyrate: Implications for intestinal function. Curr. Opin. Clin. Nutr. Metab. Care.

[64] Mohr A.E., Jäger R., Carpenter K.C., Kerksick C.M., Purpura M., Townsend J.R., West N.P., Black K., Gleeson M., Pyne D.B. (2020). The athletic gut microbiota. J. Int. Soc. Sports Nutr..

[65] Estaki M., Pither J., Baumeister P., Little J.P., Gill S.K., Ghosh S., Ahmadi-Vand Z., Marsden K.R., Gibson D.L. (2016). Cardiorespiratory fitness as a predictor of intestinal microbial diversity and distinct metagenomic functions. Microbiome.

[66] Cani P.D., Knauf C. (2016). How gut microbes talk to organs: The role of endocrine and nervous routes. Mol. Metab..

[67] Bonini J.A., Anderson S.M., Steiner D.F. (1997). Molecular Cloning and Tissue Expression of a Novel Orphan G Protein-Coupled Receptor from Rat Lung. Biochem. Biophys. Res. Commun..

[68] Xiong Y., Miyamoto N., Shibata K., Valasek M.A., Motoike T., Kedzierski R.M., Yanagisawa M. (2004). Short-chain fatty acids stimulate leptin production in adipocytes through the G protein-coupled receptor GPR41. Proc. Natl. Acad. Sci. USA.

[69] De Vadder F., Kovatcheva-Datchary P., Zitoun C., Duchampt A., Bäckhed F., Mithieux G. (2016). Microbiota-Produced Succinate Improves Glucose Homeostasis via Intestinal Gluconeogenesis. Cell Metab..

[70] Zaibi M.S., Stocker C.J., O’Dowd J., Davies A., Bellahcene M., Cawthorne M.A., Brown A.J., Smith D.M., Arch J.R. (2010). Roles of GPR41 and GPR43 in leptin secretory responses of murine adipocytes to short chain fatty acids. FEBS Lett..

[71] Nøhr M.K., Pedersen M.H., Gille A., Egerod K.L., Engelstoft M.S., Husted A.S., Sichlau R.M., Grunddal K.V., Poulsen S.S., Han S. (2013). GPR41/FFAR3 and GPR43/FFAR2 as Cosensors for Short-Chain Fatty Acids in Enteroendocrine Cells vs FFAR3 in Enteric Neurons and FFAR2 in Enteric Leukocytes. Endocrinology.

[72] Forsythe P., Sudo N., Dinan T., Taylor V., Bienenstock J. (2010). Mood and gut feelings. Brain Behav. Immun..

[73] Freestone P.P., Sandrini S.M., Haigh R.D., Lyte M. (2008). Microbial endocrinology: How stress influences susceptibility to infection. Trends Microbiol..

[74] Lyte M. (2013). Microbial Endocrinology in the Microbiome-Gut-Brain Axis: How Bacterial Production and Utilization of Neurochemicals Influence Behavior. PLoS Pathog..

[75] Lyte M. (2010). The microbial organ in the gut as a driver of homeostasis and disease. Med. Hypotheses.

[76] Macfarlane G.T., Allison C., Gibson S.A.W., Cummings J.H. (1988). Contribution of the microflora to proteolysis in the human large intestine. J. Appl. Bacteriol..

[77] Samuel B.S., Shaito A., Motoike T., Rey F.E., Backhed F., Manchester J.K., Hammer R.E., Williams S.C., Crowley J., Yanagisawa M. (2008). Effects of the gut microbiota on host adiposity are modulated by the short-chain fatty-acid binding G protein-coupled receptor, Gpr41. Proc. Natl. Acad. Sci. USA.

[78] Wong J.M.W., de Souza R., Kendall C.W.C., Emam A., Jenkins D.J.A. (2006). Colonic Health: Fermentation and Short Chain Fatty Acids. J. Clin. Gastroenterol..

[79] Al-Lahham S.H., Peppelenbosch M.P., Roelofsen H., Vonk R.J., Venema K. (2010). Biological effects of propionic acid in humans; metabolism, potential applications and underlying mechanisms. Biochim. Biophys. Acta—Mol. Cell Biol. Lipids.

[80] Cummings J.H., Pomare E.W., Branch W.J., Naylor C.P., Macfarlane G.T. (1987). Short chain fatty acids in human large intestine, portal, hepatic and venous blood. Gut.

[81] Ge H., Li X., Weiszmann J., Wang P., Baribault H., Chen J.-L., Tian H., Li Y. (2008). Activation of G Protein-Coupled Receptor 43 in Adipocytes Leads to Inhibition of Lipolysis and Suppression of Plasma Free Fatty Acids. Endocrinology.

[82] Hong Y.-H., Nishimura Y., Hishikawa D., Tsuzuki H., Miyahara H., Gotoh C., Choi K.-C., Feng D.D., Chen C., Lee H.-G. (2005). Acetate and Propionate Short Chain Fatty Acids Stimulate Adipogenesis via GPCR43. Endocrinology.

[83] Tolhurst G., Heffron H., Lam Y.S., Parker H.E., Habib A.M., Diakogiannaki E., Cameron J., Grosse J., Reimann F., Gribble F.M. (2012). Short-Chain Fatty Acids Stimulate Glucagon-Like Peptide-1 Secretion via the G-Protein-Coupled Receptor FFAR2. Diabetes.

[84] Steele R.D. (1986). Blood-brain barrier transport of the α-keto acid analogs of amino acids. Fed. Proc..

[85] Vijay N. (2014). Role of Monocarboxylate Transporters in Drug Delivery to the Brain. Curr. Pharm. Des..

[86] Maurer M. (2004). Correlation between local monocarboxylate transporter 1 (MCT1) and glucose transporter 1 (GLUT1) densities in the adult rat brain. Neurosci. Lett..

[87] Moschen I., Bröer A., Galic S., Lang F., Bröer S. (2012). Significance of Short Chain Fatty Acid Transport by Members of the Monocarboxylate Transporter Family (MCT). Neurochem. Res..

[88] Barrett E., Ross R.P., O’Toole P.W., Fitzgerald G.F., Stanton C. (2012). γ-Aminobutyric acid production by culturable bacteria from the human intestine. J. Appl. Microbiol..

[89] Lyte M. (2004). Microbial endocrinology and infectious disease in the 21st century. Trends Microbiol..

[90] Lyte M. (2011). Probiotics function mechanistically as delivery vehicles for neuroactive compounds: Microbial endocrinology in the design and use of probiotics. BioEssays.

[91] Mawe G.M., Hoffman J.M. (2013). Serotonin signalling in the gut—Functions, dysfunctions and therapeutic targets. Nat. Rev. Gastroenterol. Hepatol..

[92] Wang Z., Klipfell E., Bennett B.J., Koeth R., Levison B.S., DuGar B., Feldstein A.E., Britt E.B., Fu X., Chung Y.-M. (2011). Gut Flora Metabolism of Phosphatidylcholine Promotes Cardiovascular Disease. Nature.

[93] Koeth R.A., Wang Z., Levison B.S., Buffa J.A., Org E., Sheehy B.T., Britt E.B., Fu X., Wu Y., Li L. (2013). Intestinal microbiota metabolism of l-carnitine, a nutrient in red meat, promotes atherosclerosis. Nat. Med..

[94] Spencer M.D., Hamp T.J., Reid R., Fischer L.M., Zeisel S.H., Fodor A.A. (2011). Association between Composition of the Human Gastrointestinal Microbiome and Development of Fatty Liver with Choline Deficiency. Gastroenterology.

[95] Henao-Mejia J., Elinav E., Jin C., Hao L., Mehal W.Z., Strowig T., Thaiss C.A., Kau A.L., Eisenbarth S.C., Jurczak M.J. (2012). Inflammasome-mediated dysbiosis regulates progression of NAFLD and obesity. Nature.

[96] Ecerdá B., Epérez M., Pérez-Santiago J.D., Tornero-Aguilera J.F., Gonzalez-Soltero R., Elarrosa M. (2016). Gut Microbiota Modification: Another Piece in the Puzzle of the Benefits of Physical Exercise in Health?. Front. Physiol..

[97] Dicksved J., Halfvarson J., Rosenquist M., Järnerot G., Tysk C., Apajalahti J., Engstrand L., Jansson J.K. (2008). Molecular analysis of the gut microbiota of identical twins with Crohn’s disease. ISME J..

[98] Fransen F., van Beek A.A., Borghuis T., Meijer B., Hugenholtz F., van der Gaast-De Jongh C., Savelkoul H.F., De Jonge M.I., Faas M.M., Boekschoten M.V. (2017). The Impact of Gut Microbiota on Gender-Specific Differences in Immunity. Front. Immunol..

[99] Fontana A., Panebianco C., Picchianti-Diamanti A., Laganà B., Cavalieri D., Potenza A., Pracella R., Binda E., Copetti M., Pazienza V. (2019). Gut Microbiota Profiles Differ among Individuals Depending on Their Region of Origin: An Italian Pilot Study. Int. J. Environ. Res. Public Health.

[100] Gupta V.K., Paul S., Dutta C. (2017). Geography, ethnicity or subsistence-specific variations in human microbiome composition and diversity. Front. Microbiol..

[101] Koren O., Goodrich J.K., Cullender T.C., Spor A., Laitinen K., Bäckhed H.K., Gonzalez A., Werner J.J., Angenent L.T., Knight R. (2012). Host Remodeling of the Gut Microbiome and Metabolic Changes during Pregnancy. Cell.

[102] Salminen S., Gibson G.R., McCartney A.L., Isolauri E. (2004). Influence of mode of delivery on gut microbiota composition in seven year old children. Gut.

[103] O’Sullivan A., Farver M., Smilowitz J.T. (2015). The Influence of early infant-feeding practices on the intestinal microbiome and body composition in infants. Nutr. Metab. Insights.

[104] Nicholson J.K., Holmes E., Kinross J., Burcelin R., Gibson G., Jia W., Pettersson S. (2012). Host-Gut Microbiota Metabolic Interactions. Science.

[105] Le Chatelier E., Nielsen T., Qin J., Prifti E., Hildebrand F., Falony G., Almeida M., Arumugam M., Batto J.-M., Kennedy S. (2013). Richness of human gut microbiome correlates with metabolic markers. Nature.

[106] Sonnenburg E.D., Smits S.A., Tikhonov M., Higginbottom S.K., Wingreen N.S., Sonnenburg J.L. (2016). Diet-induced extinctions in the gut microbiota compound over generations. Nat. Cell Biol..

[107] Zinöcker M.K., Lindseth I.A. (2018). The Western Diet–Microbiome-Host Interaction and Its Role in Metabolic Disease. Nutrition.

[108] Walker A., Ince J., Duncan S.H., Webster L.M., Holtrop G., Ze X., Brown D., Stares M.D., Scott P., Bergerat A. (2010). Dominant and diet-responsive groups of bacteria within the human colonic microbiota. ISME J..

[109] Flint H.J., Scott K.P., Louis P., Duncan S. (2012). The role of the gut microbiota in nutrition and health. Nat. Rev. Gastroenterol. Hepatol..

[110] Kang S.S., Jeraldo P.R., Kurti A., Miller M.E.B., Cook M.D., Whitlock K., Goldenfeld N., Woods J.A., White B.A., Chia N. (2014). Diet and exercise orthogonally alter the gut microbiome and reveal independent associations with anxiety and cognition. Mol. Neurodegener..

[111] Bressa C., Bailen M., Pérez-Santiago J., Gonzalez-Soltero R., Pérez M., Montalvo-Lominchar M.G., Maté-Muñoz J.L., Domínguez R., Moreno D., Larrosa M. (2017). Differences in gut microbiota profile between women with active lifestyle and sedentary women. PLoS ONE.

[112] Lozupone C.A., Stombaugh J.I., Gordon J.I., Jansson J.K., Knight R. (2012). Diversity, stability and resilience of the human gut microbiota. Nature.

[113] Toor D., Wasson M.K., Kumar P., Karthikeyan G., Kaushik N.K., Goel C., Singh S., Kumar A., Prakash H. (2019). Dysbiosis Disrupts Gut Immune Homeostasis and Promotes Gastric Diseases. Int. J. Mol. Sci..

[114] Nie P., Li Z., Wang Y., Zhang Y., Zhao M., Luo J., Du S., Deng Z., Chen J., Wang Y. (2019). Gut microbiome interventions in human health and diseases. Med. Res. Rev..

[115] Guinane C.M., Cotter P.D. (2013). Role of the gut microbiota in health and chronic gastrointestinal disease: Understanding a hidden metabolic organ. Ther. Adv. Gastroenterol..

[116] Roager H.M., Hansen L.B.S., Bahl M.I., Frandsen H.L., Carvalho V., Gøbel R.J., Dalgaard M.D., Plichta D.R., Sparholt M.H., Vestergaard H. (2016). Colonic transit time is related to bacterial metabolism and mucosal turnover in the gut. Nat. Microbiol..

[117] Tierney B., Yang Z., Luber J.M., Beaudin M., Wibowo M.C., Baek C., Mehlenbacher E., Patel C.J., Kostic A.D. (2019). The Landscape of Genetic Content in the Gut and Oral Human Microbiome. Cell Host Microbe.

[118] Bayego E.S., Vila G.S., Martínez I.S. (2012). Prescripción de ejercicio físico: Indicaciones, posología y efectos adversos. Med. Clin..

[119] Munukka E., Ahtiainen J.P., Puigbó P., Jalkanen S., Pahkala K., Keskitalo A., Kujala U.M., Pietilä S., Hollmén M., Elo L. (2018). Six-Week Endurance Exercise Alters Gut Metagenome That Is not Reflected in Systemic Metabolism in Over-weight Women. Front. Microbiol..

[120] Jeukendrup A.E., Vet-Joop K., Sturk A., Stegen J.H.J.C., Senden J., Saris W.H.M., Wagenmakers A. (2000). Relationship between gastro-intestinal complaints and endotoxaemia, cytokine release and the acute-phase reaction during and after a long-distance triathlon in highly trained men. Clin. Sci..

[121] Selkirk G.A., McLellan T.M., Wright H.E., Rhind S. (2008). Mild endotoxemia, NF-κB translocation, and cytokine increase during exertional heat stress in trained and untrained individuals. Am. J. Physiol. Integr. Comp. Physiol..

[122] Yeh Y.J., Law L.Y.L., Lim C.L. (2013). Gastrointestinal response and endotoxemia during intense exercise in hot and cool environments. Graefe’s Arch. Clin. Exp. Ophthalmol..

[123] Roberts J.D., Suckling C.A., Peedle G.Y., Murphy J.A., Dawkins T.G., Roberts M.G. (2016). An Exploratory Investigation of Endotoxin Levels in Novice Long Distance Triathletes, and the Effects of a Multi-Strain Probiotic/Prebiotic, Antioxidant Intervention. Nutrition.

[124] Mailing L.J., Allen J.M., Buford T.W., Fields C.J., Woods J.A. (2019). Exercise and the Gut Microbiome: A Review of the Evidence, Potential Mechanisms, and Implications for Human Health. Exerc. Sport Sci. Rev..

[125] Cella V., Migliaccio S., Paoli A. (2020). Microbiota intestinale ed esercizio fisico: Nuova possibile area di intervento?. L’Endocrinologo.

[126] Ulrich-Lai Y., Herman J. (2009). Neural regulation of endocrine and autonomic stress responses. Nat. Rev. Neurosci..

[127] Mach N., Fuster-Botella D. (2017). Endurance exercise and gut microbiota: A review. J. Sport Health Sci..

[128] Freestone P.P., Williams P.H., Haigh R.D., Maggs A.F., Neal C.P., Lyte M. (2002). Growth Stimulation of Intestinal Commensal Escherichia coli by Catecholamines: A Possible Contributory Factor in Trauma-Induced Sepsis. Shock.

[129] Lyte M., Ernst S. (1992). Catecholamine induced growth of gram negative bacteria. Life Sci..

[130] Lyte M., Vulchanova L., Brown D.R. (2010). Stress at the intestinal surface: Catecholamines and mucosa–bacteria interactions. Cell Tissue Res..

[131] Mitchell C.M., Davy B.M., Hulver M.W., Neilson A.P., Bennett B.J., Davy K.P. (2019). Does Exercise Alter Gut Microbial Composition? A Systematic Review. Med. Sci. Sports Exerc..

[132] Queipo-Ortuño M.I., Seoane L.M., Murri M., Pardo M., Gomez-Zumaquero J.M., Cardona F., Casanueva F., Tinahones F.J. (2013). Gut Microbiota Composition in Male Rat Models under Different Nutritional Status and Physical Activity and Its Association with Serum Leptin and Ghrelin Levels. PLoS ONE.

[133] Allen J., Mailing L.J., Cohrs J., Salmonson C., Fryer J.D., Nehra V., Hale V.L., Kashyap P., White B.A., Woods J.A. (2018). Exercise training-induced modification of the gut microbiota persists after microbiota colonization and attenuates the response to chemically-induced colitis in gnotobiotic mice. Gut Microbes.

[134] Choi J.J., Eum S.Y., Rampersaud E., Daunert S., Abreu M.T., Toborek M. (2013). Exercise Attenuates PCB-Induced Changes in the Mouse Gut Microbiome. Environ. Health Perspect..

[135] Campbell S.C., Wisniewski P.J., Noji M., McGuinness L.R., Haggblom M.M., Lightfoot S.A., Joseph L.B., Kerkhof L. (2016). The Effect of Diet and Exercise on Intestinal Integrity and Microbial Diversity in Mice. PLoS ONE.

[136] Lambert J.E., Myslicki J.P., Bomhof M.R., Belke D.D., Shearer J., Reimer R.A. (2015). Exercise training modifies gut microbiota in normal and diabetic mice. Appl. Physiol. Nutr. Metab..

[137] Tung Y.-T., Hsu Y.-J., Liao C.-C., Ho S.-T., Huang C.-C., Huang W.-C. (2019). Physiological and Biochemical Effects of Intrinsically High and Low Exercise Capacities Through Multiomics Approaches. Front. Physiol..

[138] Geirnaert A., Steyaert A., Eeckhaut V., Debruyne B., Arends J., Van Immerseel F., Boon N., Van de Wiele T. (2014). *Butyricicoccus pullicaecorum*, a butyrate producer with probiotic potential, is intrinsically tolerant to stomach and small intestine conditions. Anaerobe.

[139] Steppe M., Van Nieuwerburgh F., Vercauteren G., Boyen F., Eeckhaut V., Deforce D., Haesebrouck F., Ducatelle R., Van Immerseel F. (2014). Safety assessment of the butyrate-producing Butyricicoccus pullicaecorum strain 25-3T, a potential probiotic for patients with inflammatory bowel disease, based on oral toxicity tests and whole genome sequencing. Food Chem. Toxicol..

[140] Hsu Y.J., Chiu C.C., Li Y.P., Huang W.C., Huang Y.T., Huang C.C. (2015). Effect of Intestinal Microbiota on Exercise Performance in Mice. J. Strength Cond. Res..

[141] Huang W.-C., Chen Y.-H., Chuang H.-L., Chiu C.-C. (2019). Investigation of the Effects of Microbiota on Exercise Physiological Adaption, Performance, and Energy Utilization Using a Gnotobiotic Animal Model. Front. Microbiol..

[142] Bogdanis G., Stavrinou P., Fatouros I., Philippou A., Chatzinikolaou A., Draganidis D., Ermidis G., Maridaki M. (2013). Short-term high-intensity interval exercise training attenuates oxidative stress responses and improves antioxidant status in healthy humans. Food Chem. Toxicol..

[143] Scheiman J., Luber J.M., Chavkin T., Macdonald T., Tung A., Pham L.-D., Wibowo M.C., Wurth R.C., Punthambaker S., Tierney B. (2019). Meta-omics analysis of elite athletes identifies a performance-enhancing microbe that functions via lactate metabolism. Nat. Med..

[144] Allen J.M., Mailing L.J., Niemiro G.M., Moore R., Cook M.D., White B.A., Holscher H.D., Woods J.A. (2018). Exercise Alters Gut Microbiota Composition and Function in Lean and Obese Humans. Med. Sci. Sports Exerc..

[145] Popovich D., Jenkins D.J.A., Kendall C.W.C., Dierenfeld E.S., Carroll R.W., Tariq N., Vidgen E. (1997). The Western Lowland Gorilla Diet Has Implications for the Health of Humans and Other Hominoids. J. Nutr..

[146] Frost G., Sleeth M.L., Sahuri-Arisoylu M., Lizarbe B., Cerdan S., Brody L., Anastasovska J., Ghourab S., Hankir M., Zhang S. (2014). The short-chain fatty acid acetate reduces appetite via a central homeostatic mechanism. Nat. Commun..

[147] McNeil N.I. (1984). The contribution of the large intestine to energy supplies in man. Am. J. Clin. Nutr..

[148] Okamoto T., Morino K., Ugi S., Nakagawa F., Lemecha M., Ida S., Ohashi N., Sato D., Fujita Y., Maegawa H. (2019). Microbiome potentiates endurance exercise through intestinal acetate production. Am. J. Physiol. Metab..

[149] Yan H., Diao H., Xiao Y., Li W., Yu B., He J., Yu J., Zheng P., Mao X., Luo Y. (2016). Gut microbiota can transfer fiber characteristics and lipid metabolic profiles of skeletal muscle from pigs to germ-free mice. Sci. Rep..

[150] Lahiri S., Kim H., Garcia-Perez I., Reza M.M., Martin K.A., Kundu P., Cox L.M., Selkrig J., Posma J.M., Zhang H. (2019). The gut microbiota influences skeletal muscle mass and function in mice. Sci. Transl. Med..

[151] Louis P., Flint H.J. (2009). Diversity, metabolism and microbial ecology of butyrate-producing bacteria from the human large intestine. FEMS Microbiol. Lett..

[152] Dao M.C., Everard A., Aron-Wisnewsky J., Sokolovska N., Prifti E., Verger E.O., Kayser B.D., Levenez F., Chilloux J., Hoyles L. (2016). *Akkermansia* muciniphila and improved metabolic health during a dietary intervention in obesity: Relationship with gut microbiome richness and ecology. Gut.

[153] Clarke S., Murphy E.F., Nilaweera K., Ross R., Shanahan F., O’Toole P.W., Cotter P.D. (2012). The gut microbiota and its relationship to diet and obesity. Gut Microbes.

[154] Scheepers L.E.J.M., Penders J., Mbakwa C.A., Thijs C., Mommers M., Arts I. (2014). The intestinal microbiota composition and weight development in children: The KOALA Birth Cohort Study. Int. J. Obes..

[155] Zhu L., Baker S.S., Gill C., Liu W., Alkhouri R., Baker R.D., Gill S.R. (2013). Characterization of gut microbiomes in nonalcoholic steatohepatitis (NASH) patients: A connection between endogenous alcohol and NASH. Hepatology.

[156] Cronin O., Barton W., Skuse P., Penney N.C., Garcia-Perez I., Murphy E.F., Woods T., Nugent H., Fanning A., Melgar S. (2018). A Prospective Metagenomic and Metabolomic Analysis of the Impact of Exercise and/or Whey Protein Supplementation on the Gut Microbiome of Sedentary Adults. mSystems.

[157] Kern T., Blond M.B., Hansen T.H., Rosenkilde M., Quist J.S., Gram A.S., Ekstrøm C.T., Hansen T., Stallknecht B.M. (2020). Structured exercise alters the gut microbiota in humans with overweight and obesity—A randomized controlled trial. Int. J. Obes..

[158] Durk R.P., Castillo E., Márquez-Magaña L., Grosicki G.J., Bolter N.D., Lee C.M., Bagley J.R. (2019). Gut Microbiota Composition Is Related to Cardiorespiratory Fitness in Healthy Young Adults. Int. J. Sport Nutr. Exerc. Metab..

[159] Yang Y., Shi Y., Wiklund P., Tan X., Wu N., Zhang X., Tikkanen O., Zhang C., Munukka E., Cheng S. (2017). The Association between Cardiorespiratory Fitness and Gut Microbiota Composition in Premenopausal Women. Nutrition.

[160] Cho I., Yamanishi S., Cox L., Methé B.A., Zavadil J., Li K., Gao Z., Mahana D., Raju K., Teitler I. (2012). Antibiotics in early life alter the murine colonic microbiome and adiposity. Nat. Cell Biol..

[161] Turnbaugh P.J., Ley R.E., Mahowald M.A., Magrini V., Mardis E.R., Gordon J.I. (2006). An obesity-associated gut microbiome with increased capacity for energy harvest. Nature.

[162] Morita E., Yokoyama H., Imai D., Takeda R., Ota A., Kawai E., Hisada T., Emoto M., Suzuki Y., Okazaki K. (2019). Aerobic Exercise Training with Brisk Walking Increases Intestinal Bacteroides in Healthy Elderly Women. Nutrition.

[163] Zhao X., Zhang Z., Hu B., Huang W., Yuan C., Zou L. (2018). Response of Gut Microbiota to Metabolite Changes Induced by Endurance Exercise. Front. Microbiol..

[164] Clavel T., Lepage P., Charrier C. (2014). The family Coriobacteriaceae. The Prokaryotes: Actinobacteria.

[165] Keohane D.M., Woods T., O’Connor P., Underwood S., Cronin O., Whiston R., O’Sullivan O., Cotter P., Shanahan F., Molloy M.G. (2019). Four men in a boat: Ultra-endurance exercise alters the gut microbiome. J. Sci. Med. Sport.

[166] Hampton-Marcell J.T., Eshoo T.W., Cook M.D., Gilbert J.A., Horswill C.A., Poretsky R. (2020). Comparative Analysis of Gut Microbiota Following Changes in Training Volume Among Swimmers. Int. J. Sports Med..

[167] Everard A., Lazarevic V., Derrien M., Girard M., Muccioli G.G., Neyrinck A.M., Possemiers S., Van Holle A., François P., de Vos W.M. (2011). Responses of Gut Microbiota and Glucose and Lipid Metabolism to Prebiotics in Genetic Obese and Diet-Induced Leptin-Resistant Mice. Diabetes.

[168] Karlsson C.L., Önnerfält J., Xu J., Molin G., Ahrné S., Thorngren-Jerneck K. (2012). The Microbiota of the Gut in Preschool Children with Normal and Excessive Body Weight. Obesity.

[169] Lim M.Y., Rho M., Song Y.M., Lee K., Sung J., Ko G. (2014). Stability of gut enterotypes in Korean monozygotic twins and their association with biomarkers and diet. Sci. Rep..

[170] Wu G.D., Chen J., Hoffmann C., Bittinger K., Chen Y.Y., Keilbaugh S.A., Bewtra M., Knights D., Walters W.A., Knight R. (2011). Linking Long-Term Dietary Patterns with Gut Microbial Enterotypes. Science.

[171] Noguera-Julian M., Rocafort M., Guillén Y., Rivera J., Casadellà M., Nowak P., Hildebrand F., Zeller G., Parera M., Bellido R. (2016). Gut Microbiota Linked to Sexual Preference and HIV Infection. EBioMedicine.

[172] Liu Z., Liu H.-Y., Zhou H., Zhan Q., Lai W., Zeng Q., Ren H., Xu D. (2017). Moderate-Intensity Exercise Affects Gut Microbiome Composition and Influences Cardiac Function in Myocardial Infarction Mice. Front. Microbiol..

[173] Matziouridou C., Marungruang N., Nguyen T.D., Nyman M., Fåk F. (2016). Lingonberries reduce atherosclerosis in *Apoe*^−/−^ mice in association with altered gut microbiota composition and improved lipid profile. Mol. Nutr. Food Res..

[174] Haro C., Montes-Borrego M., Rangel-Zuñiga O.A., Alcala-Diaz J.F., Gómez-Delgado F., Pérez-Martínez P., Delgado-Lista J., Quintana-Navarro G.M., Tinahones F.J., Landa B.B. (2016). Two Healthy Diets Modulate Gut Microbial Community Improving Insulin Sensitivity in a Human Obese Population. J. Clin. Endocrinol. Metab..

[175] Wu F., Guo X., Zhang J., Zhang M., Ou Z., Peng Y. (2017). Phascolarctobacterium faecium abundant colonization in human gastrointestinal tract. Exp. Ther. Med..

[176] He X., Ding L., Su W., Ma H., Huang H., Wang Y., Ren H. (2018). Distribution of endotoxins in full scale pharmaceutical wastewater treatment plants and its relationship with microbial community structure. Water Sci. Technol..

[177] Lennard K., Dabee S., Barnabas S.L., Havyarimana E., Blakney A., Jaumdally S.Z., Botha G.R., Mkhize N.N., Bekker L.-G., Lewis D.A. (2018). Microbial Composition Predicts Genital Tract Inflammation and Persistent Bacterial Vaginosis in South African Adolescent Females. Infect. Immun..

[178] Liang R., Zhang S., Peng X., Yang W., Xu Y., Wu P., Chen J., Cai Y., Zhou J. (2019). Characteristics of the gut microbiota in professional martial arts athletes: A comparison between different competition levels. PLoS ONE.

[179] O’Donovan C.M., Madigan S.M., Garcia-Perez I., Rankin A., Sullivan O.O., Cotter P. (2020). Distinct microbiome composition and metabolome exists across subgroups of elite Irish athletes. J. Sci. Med. Sport.

[180] Hwang N., Eom T., Gupta S.K., Jeong S.-Y., Jeong D.-Y., Kim Y.S., Lee J.-H., Sadowsky M.J., Unno T. (2017). Genes and Gut Bacteria Involved in Luminal Butyrate Reduction Caused by Diet and Loperamide. Genes.

[181] Jang L.G., Choi G., Kim S.W., Kim B.Y., Lee S., Park H. (2019). The combination of sport and sport-specific diet is associated with characteristics of gut microbiota: An observational study. J. Int. Soc. Sports Nutr..

[182] Marttinen M., Ala-Jaakkola R., Laitila A., Lehtinen M.J. (2020). Gut Microbiota, Probiotics and Physical Performance in Athletes and Physically Active Individuals. Nutrients.

[183] Diether N.E., Willing B.P. (2019). Microbial Fermentation of Dietary Protein: An Important Factor in Diet–Microbe–Host Interaction. Microorganisms.

[184] Portune K.J., Benítez-Páez A., del Pulgar E.M.G., Cerrudo V., Sanz Y. (2017). Gut microbiota, diet, and obesity-related disorders—The good, the bad, and the future challenges. Mol. Nutr. Food Res..

[185] Macfarlane G.T., Macfarlane S. (2012). Bacteria, Colonic Fermentation, and Gastrointestinal Health. J. AOAC Int..

[186] Lamprecht M., Frauwallner A. (2012). Exercise, Intestinal Barrier Dysfunction and Probiotic Supplementation. Acute Top. Sport Nutr..

[187] Laureto L.M.O., Cianciaruso M., Samia D.S.M. (2015). Functional diversity: An overview of its history and applicability. Nat. Conserv..

[188] Contrepois K., Wu S., Moneghetti K.J., Hornburg D., Ahadi S., Tsai M.-S., Metwally A.A., Wei E., Lee-McMullen B., Quijada J.V. (2020). Molecular Choreography of Acute Exercise. Cell.

[189] Milani C., Ticinesi A., Gerritsen J., Nouvenne A., Lugli G.A., Mancabelli L., Turroni F., Duranti S., Mangifesta M., Viappiani A. (2016). Gut microbiota composition and Clostridium difficile infection in hospitalized elderly individuals: A metagenomic study. Sci. Rep..

[190] Robinson S.M., Reginster J.Y., Rizzoli R., Shaw S.C., Kanis J.A., Bautmans I., Bischoff-Ferrari H., Bruyère O., Cesari M., Dawson-Hughes B. (2017). Does nutrition play a role in the prevention and management of sarcopenia?. Clin. Nutr..

[191] Landi F., Marzetti E., Martone A.M., Bernabei R., Onder G. (2013). Exercise as a remedy for sarcopenia. Curr. Opin. Clin. Nutr. Metab. Care.

[192] Ticinesi A., Lauretani F., Milani C., Nouvenne A., Tana C., Del Rio D., Maggio M., Ventura M., Meschi T. (2017). Aging Gut Microbiota at the Cross-Road between Nutrition, Physical Frailty, and Sarcopenia: Is There a Gut–Muscle Axis?. Nutrition.

[193] Ticinesi A., Lauretani F., Tana C., Nouvenne A., Ridolo E., Meschi T. (2019). Exercise and immune system as modulators of intestinal microbiome: Implications for the gut-muscle axis hypothesis. Exerc. Immunol. Rev..

